# European In-Situ Snow Measurements: Practices and Purposes

**DOI:** 10.3390/s18072016

**Published:** 2018-06-22

**Authors:** Roberta Pirazzini, Leena Leppänen, Ghislain Picard, Juan Ignacio Lopez-Moreno, Christoph Marty, Giovanni Macelloni, Anna Kontu, Annakaisa von Lerber, Cemal Melih Tanis, Martin Schneebeli, Patricia de Rosnay, Ali Nadir Arslan

**Affiliations:** 1 Finnish Meteorological Institute (FMI), FI-00101 Helsinki, Finland; leena.leppanen@fmi.fi (L.L.); anna.kontu@fmi.fi (A.K.); Annakaisa.von.Lerber@fmi.fi (A.v.L.); Cemal.Melih.Tanis@fmi.fi (C.M.T.); Ali.Nadir.Arslan@fmi.fi (A.N.A.); 2 UGA, CNRS, Institut des Géosciences de l’Environnement (IGE), UMR 5001, F-38041 Grenoble, France; ghislain.picard@univ-grenoble-alpes.fr; 3 Instituto Pirenaico de Ecología, CSIC, 50059 Zaragoza, Spain; nlopez@ipe.csic.es; 4 WSL Institute for Snow and Avalanche Research (SLF), CH-7260 Davos Dorf, Switzerland; marty@slf.ch (C.M.); schneebeli@slf.ch (M.S.); 5 CNR, Institute of Applied Physics “Nello Carrara” (IFAC), 50019 Sesto Fiorentino, Italy; g.macelloni@ifac.cnr.it; 6 European Centre for Medium Range-Weather Forecasts (ECMWF), Reading RG2 9AX, UK; Patricia.Rosnay@ecmwf.int

**Keywords:** snow properties, in-situ measurements, instruments

## Abstract

In-situ snow measurements conducted by European institutions for operational, research, and energy business applications were surveyed in the framework of the European Cooperation in Science and Technology (COST) Action ES1404, called “A European network for a harmonised monitoring of snow for the benefit of climate change scenarios, hydrology, and numerical weather prediction”. Here we present the results of this survey, which was answered by 125 participants from 99 operational and research institutions, belonging to 38 European countries. The typologies of environments where the snow measurements are performed range from mountain to low elevated plains, including forests, bogs, tundra, urban areas, glaciers, lake ice, and sea ice. Of the respondents, 93% measure snow macrophysical parameters, such as snow presence, snow depth (HS), snow water equivalent (SWE), and snow density. These describe the bulk characteristics of the whole snowpack or of a snow layer, and they are the primary snow properties that are needed for most operational applications (such as hydrological monitoring, avalanche forecast, and weather forecast). In most cases, these measurements are done with manual methods, although for snow presence, HS, and SWE, automatized methods are also applied by some respondents. Parameters characterizing precipitating and suspended snow (such as the height of new snow, precipitation intensity, flux of drifting/blowing snow, and particle size distribution), some of which are crucial for the operational services, are measured by 74% of the respondents. Parameters characterizing the snow microstructural properties (such as the snow grain size and shape, and specific surface area), the snow electromagnetic properties (such as albedo, brightness temperature, and backscatter), and the snow composition (such as impurities and isotopes) are measured by 41%, 26%, and 13% of the respondents, respectively, mostly for research applications. The results of this survey are discussed from the perspective of the need of enhancing the efficiency and coverage of the in-situ observational network applying automatic and cheap measurement methods. Moreover, recommendations for the enhancement and harmonization of the observational network and measurement practices are provided.

## 1. Introduction

Snow is considered the largest gap in the current knowledge of the global water budget [1]. It is a critical component of the Earth’s ecosystem, it is very sensitive to climate change [2], and causes strong climate feedbacks. In-situ snow observations are sparse; vast snow covered areas are hardly accessible, and therefore snow monitoring mostly relies on satellite snow observations. However, these have several limitations, which depend on the parameters that we plan to investigate. For instance, snow cover maps mainly rely on optical sensors, which are affected by the presence of clouds; active microwave sensors have demonstrated their capability in mapping dry/wet status of snow, but do not have a high temporal coverage; snow water equivalent (SWE) can be derived by microwave passive sensors, which have a coarse resolution (i.e., tens of kilometres), have a limited operability on mountain regions, and have a high uncertainty for high SWE values (i.e., higher than 120 mm). In the ongoing climate change, the snow cover extent is decreasing [3], modifying the Northern Hemisphere atmospheric circulation [4]. Thus, snow measurements are and will be more and more crucial for freshwater management, mitigation of climate changes, adaptation to new climate conditions, and risk assessments (such as avalanches and floods). Lastly, the evolution of the snow cover is a major concern in some specific mountainous locations (e.g., alpine regions), where the economic activity depends on it (e.g., [5,6]). Snow can indeed be a critical resource for tourism (e.g., ski resorts), water supply, and hydropower energy.

The advances in the modelling of the snow-electromagnetic interaction (e.g., [7,8,9,10]) and in the observational capabilities of the satellite-based sensors (e.g., the sensors on board ESA Sentinel missions) have pushed the development of new in-situ instrumentation, which are able to provide suitable reference and ground-truth data for the validation of snow satellite products and of earth system models. In particular, instruments that can measure snow microstructure properties in the field have appeared, such as the IceCube [11], ASSSAP [12], or the SnowMicroPen [13], although they are available only for a few expert users. The testing of physically-based snow models and retrieval algorithms requires, on one hand, that the basic snow properties (such as snow depth, SWE, snow density) are measured with better precision and temporal resolution and, on the other hand, that new snow properties (such as, the surface roughness and snow correlation length) become observable. Having this objective, different institutions and research teams have developed different snow measurement practices and instrumentations that are customized to their purposes. While these efforts constitute significant steps forward in the understanding of snow physical processes, the lack of coordination in the measurements procedure and measured parameters limits the capability to share knowledge and data in the community. It is worth noting that, besides the new mentioned measurements capabilities, the instruments and methodologies for measuring basic snow parameters from ground (e.g., snow density, snow depth, SWE) also strongly differ among different institutions.

Indeed, while the International Classification for Seasonal Snow on the Ground [14] provides the definition of most of the observable snow properties, the classification of snow grain shapes, and a guideline to perform traditional snowpit measurements, it does not include a systematic description of the instruments that are used for snow monitoring. A recent review of the in-situ measurement techniques and devices that are used to determine the physical properties of the seasonal snowpack was done by Kinar and Pomeroy [15]. Their historical perspective illustrates the development and state-of-the-art of the field, with a focus on the practices that are applied in North America and Canada, which, however, do not entirely correspond to the current practices that are in use by European institutions. Moreover, the review by Kinar and Pomeroy [15] does not address the effective use of the devices.

In order to fill this gap and harmonize the snow monitoring procedure, the European Cooperation in Science and Technology (COST) promoted the Action ES1404 called “Harmosnow” (“A European network for a harmonised monitoring of snow for the benefit of climate change scenarios, hydrology, and numerical weather prediction”). The aim of the action is to coordinate the effort of harmonizing the snow monitoring practices, by promoting new observing strategies, bringing together different communities, facilitating data transfer, upgrading and enlarging knowledge through networking, exchange and training, and linking them to activities in international agencies and global networks. 

One of the first activities of Harmosnow was to carry out a survey to obtain an updated picture of the existing variety of snow measurement practices and instrumentations in use by the European institutions. The survey collected a compilation of the measured snow properties and associated measurement techniques that are applied by the participating European countries for a large variety of applications. This paper aims to summarize the results of this survey, providing an overview on the European snow measurements that are carried out for a large variety of applications. Synthesis from the overview enables us to draw recommendations on the best measurement practices and on strategies to increase the effectiveness and the extension of the snow monitoring network.

## 2. European Survey on Snow Measurement Practices and Applied Instrumentation

A survey on the measured snow properties and applied instrumentation was carried out among the European institutions through a questionnaire that was designed with the contribution of several partners of Harmosnow with experience on snow observations. The questionnaire only addressed general information, as each respondent was asked to fill it in just once.

To get unambiguous answers, despite the absence of standard terminology for many snow properties, and even more importantly for devices or techniques, most of the questions were formulated as multiple choices. This required an investigation on the existing measurable snow properties and measurement instruments. Snow properties were grouped into five categories: snow macrophysical properties, snow microphysical properties, snow electromagnetic properties, precipitating and suspended snow, and snow composition. The measurable parameters that were associated to the snow macrophysical properties, microphysical properties, electromagnetic properties, solid precipitation and snow composition, as well as the existing instruments to measure them, are listed and defined in Appendix A in Table A1, Table A2, Table A3, Table A4 and Table A5, respectively. Table A6 in Appendix B is complementary to the tables in Appendix A, as it includes a description of the instruments that are applied to measure or derive the snow parameters.

Snow macrophysical properties describe the bulk characteristics of the whole snowpack or of a snow layer. Some of them, such as snow presence, snow depth (HS), and SWE, are the most important input information for hydrological and numerical weather prediction models, and therefore, they have the most established and wide spread observational network. Others (such as snow hardness, penetrability of snow surface, and snow strength) are mostly measured for snow avalanche forecast, thus in mountain regions only.

Snow microphysical properties describe the characteristics of the snow microstructure, such as snow grain shape, snow specific surface area, snow correlation length, etc. (a comprehensive list of these properties is given in Table A2). The estimation of these properties is needed, for instance, to simulate the interaction of the snow with electromagnetic radiation, with the purpose of developing algorithms to retrieve important variables, such as SWE or near-surface snow microstructure properties from the satellite observations. The snow microphysical properties are also utilized in the snow physical models that support avalanche forecasting [16,17]. 

Snow electromagnetic properties characterize the interaction of snow with the electromagnetic radiation. In the visible spectral region, the snow albedo controls the amount of solar energy that is reflected back to the atmosphere and is absorbed by the snowpack, being thus a key parameter for the snow energy and mass budget, and in particular, for meltwater runoff estimations and forecasts. In the near-infrared wavelengths, the albedo of snow is lower and strongly depends on the microstructural characteristics of the near-surface layer. Thus, the snow infrared albedo is applied to retrieve the snow microstructural properties (e.g., [18]). In the microwave spectral region, the signal that is captured by radiometers and radars over snow-covered surfaces (i.e., the electromagnetic radiation emitted or reflected by it) depends on SWE, snow microstructure, melting/freezing state, and background (e.g., soil, rocks, shrubs, etc.). By using appropriate retrieval algorithms, these variables can be hence derived remotely (e.g., [19,20,21,22]). Note that the ground-based instruments that are used to measure snow electromagnetic properties are similar (as frequencies, polarizations, etc.) to those that are installed on board satellites, and that these can be used to calibrate and validate satellite-derived snow data and extend the analysis over a broader area.

Solid precipitation has a variety of forms, including snowflakes, snow crystals, and graupel (see definitions in the Global Cryosphere Watch site: http://globalcryospherewatch.org/about/about_solidprecip.html). The amount of precipitation is an essential input for all of the models that simulate the snow cover and produce forecasts of weather, avalanches, melt water amount, and floods. Drifting and blowing snow is horizontally transported by the wind through saltation (i.e., in periodic contact with and directly above the snow surface) [23]. The snow crystals that are transported by the wind have a different shape and smaller size than the solid precipitation [24], as during the saltation process, they break and sublimate. 

In our survey, the physical-chemical composition of snow mainly concerns light absorbing impurities as well as water and heavy metal isotopes. Snow impurities affect the snow albedo, and therefore the absorption of solar energy by the snowpack. The isotopic composition of snow is related to the atmospheric conditions occurring during the snow formation in the atmosphere, as well as to the degree of snow metamorphism.

In the questionnaire, for each category of snow property, the respondents were first asked to specify if any of the snow properties that were listed in that category were measured or not. Only if the response was “yes”, the questions concerning that category were asked. Each snow property was associated with one of the instruments that were available to measure it, thus, respondents needed to select all of the combinations of property/instrument that they apply in the field. This strategy facilitated the respondents, as no free text was required (except if the applied property/instrument combination was missing from the list) and it also ensured a consistent terminology, which was necessary for the interpretation and analysis of the results. The questionnaire also included a question to find out whether a written protocol (published or unpublished, written in any language) is used when doing the snow measurements, as well as questions to identify the landscape where the measurements are carried out and the purpose of the measurements. The questionnaire is reported in Appendix C. It was conducted between December 2015 and November 2017 and it was distributed among the COST member countries, through their national representatives within Harmosnow, who were invited to send it also to their national contacts (colleagues doing snow measurements, and snow specialists in the private sector). As there was no significant coordination of national snow measurements in any of the countries that were participating in the survey, the distribution of the questionnaire mostly relied on personal effort and on the contact network of the individual Harmosnow representatives. No instructions were given concerning the profile or professional level of the respondents, and it was not defined whether the answers should represent the measurement practices that are applied by a single person, a group, or the whole affiliated institution. Therefore, the collected answers are largely heterogeneous in terms of institutional representativeness. We did not apply filters or weight to the answers to balance these differences of representativeness, but we discussed the answers case by case when needed, and we accounted for the potential limitations in the representativeness in our conclusions. All of the received answers were analysed manually (i.e., the presented statistical calculations were not automatically generated by the software that was used to create the questionnaire).

## 3. Results

This section is divided by subheadings. It provides a concise and precise description of the results of the questionnaire and their interpretation.

### 3.1. Participating Countries and Institutions

**Figure 1:**
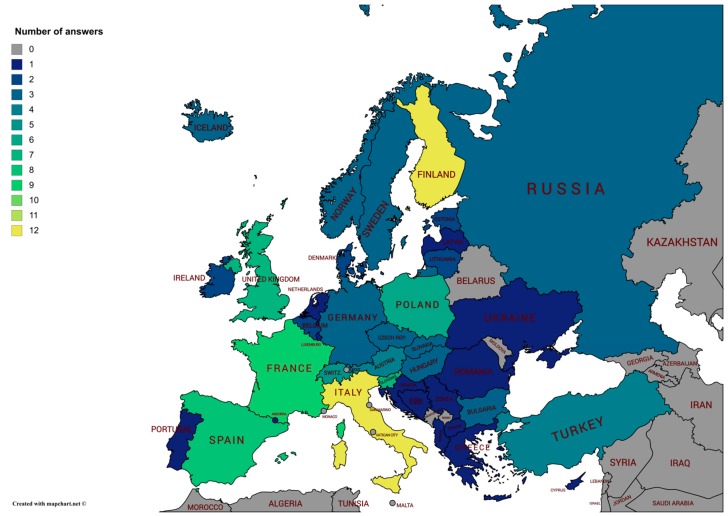
Map of Europe showing the countries that participated in the survey, and the number of answers per country (through the color categories).

The questionnaire was answered by 125 participants from 99 operational bodies, research institutions, and energy providers, from 38 European countries (Table A7 in Appendix D). Generally, most of the answers were given by those countries that provide weather-related services through several institutions, that are possibly distributed in different areas of the national territory, or that promote snow-related research in various universities, research institutes, or research groups. For most of the participating countries, at least one answer was given by the institution that provides the national weather service. Exceptions were Andorra, Greece, Luxembourg, and The Netherlands, from which the only received answers were from the Snow and Mountain Research Center (Andorra), the University of Thessaly (Greece), the Agricultural Service (Luxembourg), and the Utrecht University (The Netherland). As illustrated in Figure 1, the largest number of answers was from Finland and Italy (twelve answers each, together corresponding to about 19% of the total). In the case of Finland, the respondents were mostly from the Finnish Meteorological Institute, which includes the national operational services for weather, aviation, marine, and terrestrial traffic, as well as a research division with a lot of focus on polar areas. In the case of Italy, most of the answers (9/12) were from operational services, eight of them were regional and one was nation-wide (the Technical Centre for Meteorology of the Italian Air Force). A relatively large number of answers were given also from France, Spain, the United Kingdom, Slovenia, and Poland (nine, eight, seven, seven, and six answers, respectively). In those countries, most of the answers came from research institutes or universities, and except for Slovenia, one answer was provided by the respective national meteorological offices. Other answers were given by water management agencies (from Spain and the U.K.), environment protection agencies (from Spain, Slovenia, and the U.K.), avalanche/mountain rescue services (from Slovenia and the U.K.), and electrical companies (from France). Switzerland, Austria, Turkey, Bulgaria, Czech Republic, Hungary, Germany, Iceland, Norway, Russia, Slovakia, and Sweden provided three to five answers, generally distributed between operational services (related to weather, hydrology, and avalanches) and research institutions, with the addition of an energy company from Iceland. The twenty-two remaining countries provided only one or two answers, generally from the national weather service. Only in the case of the Netherlands and Greece, the single answer was given by a university. While not all of the European research and operational institutions that are involved in snow measurements responded to the survey, we believe, based on the numbers that were obtained and the qualification of the respondents, that it well represents the status of the methodologies that are adopted for snow monitoring at a European level.

### 3.2. Purpose of the Measurements and Measurement Environment

**Figure 2:**
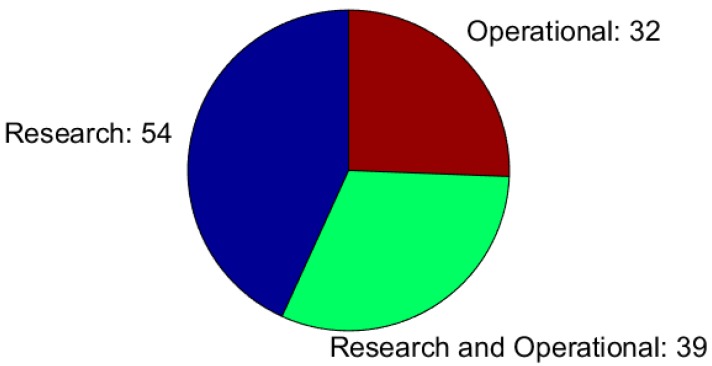
Purpose of the snow measurements, and number of respondents selecting them. The width of each slice of the pie represents the fraction of responses declaring ‘Research’, ‘Operational’, or both.

Figure 2 illustrates the fraction of responses declaring research only, operational only, or both research and operational purposes of the snow measurements. The research purpose was dominating, with the largest percentage of responses (43%) attributing the exclusive purpose of the snow measurements to research. However, responses from operational oriented institutions (71 responses) were more numerous than the responses from the research only institutions (54 responses), but 55% of them had also research objectives. On the contrary, research institutions generally did not have any operational purposes. 

**Figure 3:**
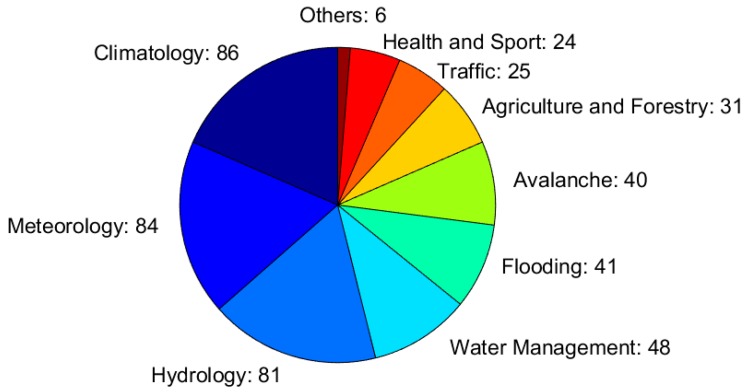
Application areas of the snow measurements, and the number of respondents selecting them. The width of each slice corresponds to the fraction each application area has, relative to the total number of the application areas reported (i.e., the sum of the numbers given in the figure).

The application areas of snow measurements are illustrated in Figure 3. The respondents could select more than one of the listed application areas, and eventually add extra applications (in the option ‘other’), if relevant. Hence, the total number of selected application areas is the sum of the numbers that are reported in Figure 3. Of the respondents, 86 (69% of the total number of selected application areas) listed ‘climatology’ among their application areas, 84 respondents (67% of the total) listed ‘meteorology’, and 91 respondents (65% of the total) listed ‘hydrology’, although these may not be the main scope of the operational oriented institutions (in the questionnaire, it was not asked to provide a hierarchic order of the relevance of the application areas). Specific applications, such as avalanche risk forecast, water management, or agriculture and forestry, are often handled in dedicated institutions, thus, the number of respondents selecting those areas were necessarily lower than the number of respondents selecting more general purposes. Among these specific application areas, the most frequent one was water management (48 respondents, corresponding to 38% of the total), followed by flooding forecast (41 respondents), avalanche risk forecast, agriculture and forestry, traffic, and health and sport. It is also worth mentioning that two participants stated ‘energy production’ in the option ‘other’, and other four stated ‘permafrost’, ‘environment protection’, ‘geodesy and gravity’, and ‘remote sensing of temperature and humidity’, respectively.

**Figure 4:**
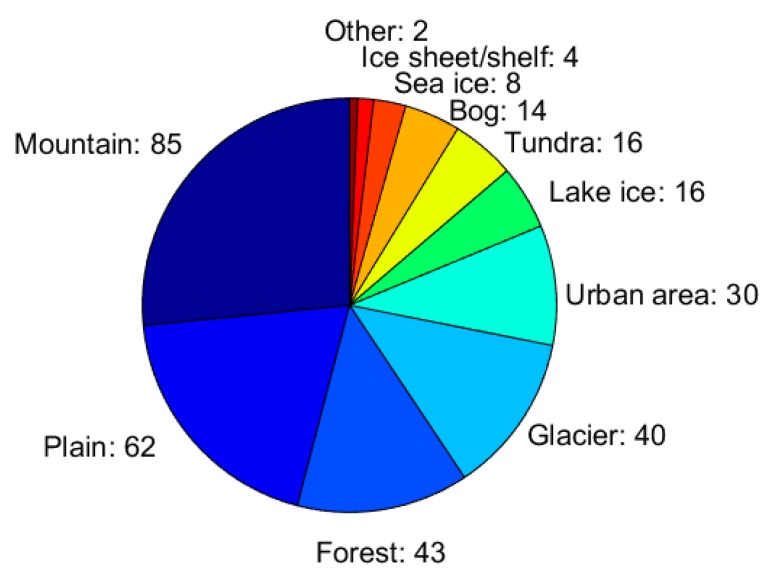
Types of measurement sites reported by the respondents. The width of each slice corresponds to the fraction each site type has, relative to the total number of site types reported (i.e., the sum of the numbers given in the figure).

Figure 4 illustrates the occurrence of measurement sites typologies. As in the case of the application areas, the respondents could select more than one of the listed site typologies and eventually add extra ones (in the option other). The multi-choice option was allowed because a single respondent/institution may perform measurements in different site types, and because a single site may belong to more than one typology (e.g., a forest covered mountain site could have been classified as ‘mountain’ and ‘forest’, an Alpine glacier as ‘mountain’ and ‘glacier’, etc.). The total number of the selected site typologies was the sum of the numbers that are reported in Figure 4. The figure clearly demonstrated the diversity of the snow conditions across Europe. In Southern and Western Europe, seasonal snow is present mostly over the mountains and only occasionally at sea level, while in Northern and in the more continental Eastern Europe, the occurrence of seasonal snow at the sea level is more frequent. Mountains are present in all of the European countries, except the Netherlands, Denmark (without Greenland), Estonia, Latvia, Lithuania, Belarus, Moldova, Malta, and Finland (if we exclude the area close to the Norwegian border) and are covered by seasonal snow, thus quite expectedly, they were the most frequent types of environment (selected by 85 respondents, corresponding to 68% of the total). On the other hand, the snow measurements sites are preferably chosen on flat open areas, so that the observations are not affected by nearby obstacles or slopes that would cause local redistribution or heterogeneity, compromising the representativeness of the site or the simple interpretation of the data. This explains why plains (open areas) had the second largest occurrence among the selected environment types. The landscape in the Northern and Eastern Europe is characterized by a large presence of forests, while in Southern and Western Europe, forests are concentrated into the mountainous areas, and the landscape is more urban and is dominated by cultivated fields. Glaciers are present in 13 of the 38 countries that were participating in the questionnaire. Frozen lakes and sea ice, over which snow could accumulate, cover significant areas only in Northern Europe (Scandinavia and northern Russia) during winter. Tundra is the characteristic landscape of the subpolar Europe (north of Scandinavia, Iceland, and North Russia), while bogs are present in a few European countries (Czech Republic, Estonia, Finland, Germany, Ireland, Norway, Sweden, Switzerland, and the United Kingdom). Some of the research institutions that were participating in the survey also have field measurement activities over ice sheets and ice shelves (four responses). These institutions apply their snow measurement practices also in the polar regions outside Europe (e.g., in Antarctica), implying a natural transfer of the gained knowledge that is beneficial for the measurements that are carried out in the European polar areas (Greenland, Svalbard, Northern Scandinavia, Northern Russia, and, in some respects, the high Alps). The category ‘other’ in Figure 4 included airports and some laboratories that produce artificial snow. These differences in the environment types obviously affect the measured snow properties and the applied measurement techniques.

### 3.3. Measured Properties and Applied Instrumentation

Kinar and Pomoroy [15] provided a classification of the instrumentation, distinguishing between ‘portable’ or ‘stationary’, ‘invasive’ or ‘non-invasive’, and ‘active’ or ‘passive’. Non-invasive measurements, which do not modify the snowpack, can be more easily automated than the invasive measurements, and can be made stationary for long term monitoring. Active or passive measurements refer to devices that send and receive (active), or only receive (passive) electromagnetic or sound waves. Here, we also classified the instruments distinguishing between ‘manual’ and ‘automatic’, as this classification better illustrates the possible application of the measurements: sustainable observations for monitoring usually require automatic devices, which also imply that the instruments are stationary and non-invasive. Among the manual instruments, we also distinguished between ‘manual without electronics’ and ‘manual with electronics’, the latter having the potential of being further developed into automatic devices (when they are operated remotely and not in a snowpit). We also distinguished the devices that are unique, developed by a single group or company, and are usually known by their name (written in italics in the plots and the tables), versus the generic techniques or devices which obviously encompass a larger diversity than the former ones. All of the instruments that were listed in the survey and are cited in the present paper are described in Appendix B in Table A6, with references to the literature illustrating their application. Table A6 also includes the classification of the instruments and the snow properties that are measured by/derived from each of them.

#### 3.3.1. Snow Macrophysical Properties

**Figure 5:**
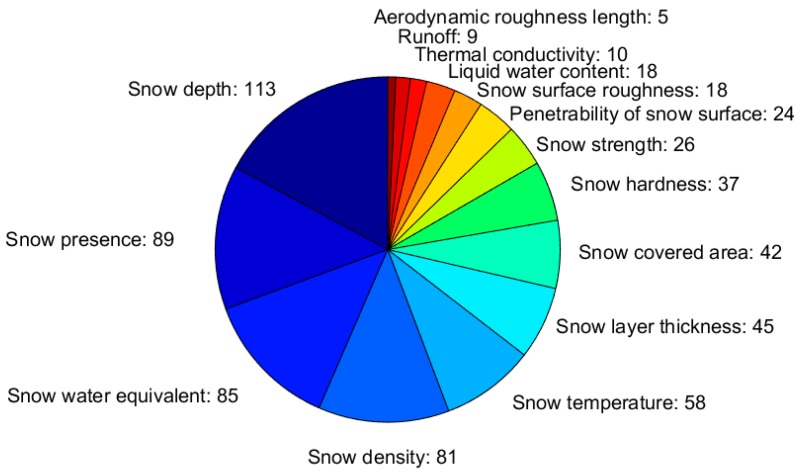
Measured snow macrophysical properties. The width of each slice corresponds to the fraction each macrophysical property has relative to the total number of reported macrophysical properties (i.e., the sum of the numbers given in the figure).

**Figure 6:**
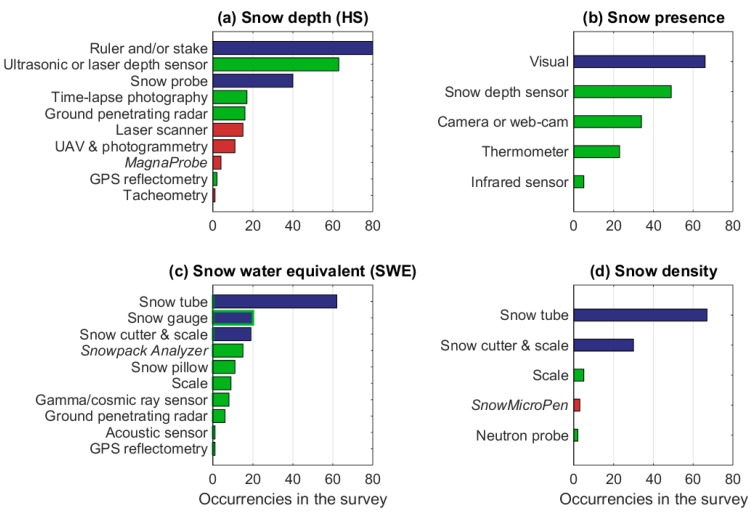
Occurrences of the applied instruments to measure snow macrophysical properties, such as snow depth (**a**), snow presence (**b**), snow water equivalent (**c**), and snow density (**d**). Different colours denote manual instrument without electronics (blue), manual instrument with electronics (red), manual instrument without electronic or fully automatic depending on the version of the instrument (blue with green contour), and fully automatic instrument (green).

Snow macrophysical properties are the most commonly measured, as 117 respondents (93% of the total) measure one or (more often) several of them. Figure 5 illustrates how frequently each macrophysical property is measured among the respondents, with respect to the total number of measured macrophysical properties. HS is the most measured property (measured by 113 respondents), followed by snow presence, SWE, and snow bulk density. These four properties provide the basic knowledge on the snowpack, and the occurrences of the measurement techniques that are applied to measure them are illustrated in Figure 6 (see Table A6 for a description of the instruments). It should be pointed out that, in many cases, multiple instruments and techniques are often used, as it occurs in particular for HS and SWE. This means that more instruments are simultaneously deployed in the same location (for instrument inter-comparison and calibration), or that different instruments are used by the same respondent in different measurement campaigns/stations/time periods. 

The most common measurement methods for HS, snow presence (snow on/off), SWE, and snow density are not automated. Often, however, both manual and automatic methods are applied by the same respondents, highlighting the complementarity of the techniques. Indeed, although 74% of the respondents measuring snow presence do visual observations, 78% of them perform also automatic measurements, retrieving snow presence from, for example, snow depth sensors, camera, web-cam, or indirectly estimating it from the thermometer automatic record. About 73% of the respondents measuring HS apply manual ruler and/or stakes. In the questionnaire, snow stakes and rulers were given as two distinct instrument options to measure the HS. However, their distinction is not always clear, especially in the case of the stationary installations. Therefore, in Figure 6b, we merged the responses in which either one of them or both of them were selected, the latter corresponding to about half of the cases. About 57% of the respondents measuring HS do automatic ultrasonic or laser depth sensors. Of these, 82% utilize also one or more manual methods, such as rulers, stakes, and snow probes. Although it was not specifically mentioned among the possible HS measurement methods, GPS reflectometry (see definition in Table A6) is an emerging technique that takes advantage of the large number of GPS satellites as a free timestamped electromagnetic source, and it is used by the Slovakian Institute of Hydrology and the Geodetic Institute of Slovenia. The latter institute also applies tacheometry to measure HS and the snow covered area over slopes. Another interesting approach that is adopted by the Federal Hydrometeorological Institute of Bosnia and Herzegovina is to infer snow cover bulk properties from the soil measurements (mainly temperature). This approach cannot provide accurate quantitative estimations of HS or SWE, nevertheless, it allows the detection of snow presence and a rough estimation of HS in a very cheap and automatic way, and therefore it is potentially applicable over large areas. An example of the relationship between snow presence and soil temperature is given by Mackiewicz [25].

A large number of techniques are applied to measure the SWE (Figure 6c). While most of them measure the SWE directly targeting the entire snowpack, the snow gauge measures the daily SWE of the new snow. From the cumulative snow gauge measurements, the SWE of the snowpack can be obtained with assumptions on (or independent estimations of) snow melting and sublimation, hence the accuracy of the measurement is lower than in the case of the other techniques. For the estimation of HS, snow presence, and SWE, several automatic instruments are available, some of them quite sophisticated (such as gamma and cosmic ray sensors, or ground penetrating radars), but others quite mature in technology and easily available and affordable (such as ultrasonic depth sensor for HS, camera or web-cam for snow presence, snow pillow for SWE, and automated snow gauge for daily SWE of new snow). While the old snow gauges were manual, hence requiring the manual emptying of the gauge to measure the accumulated snow volume, in many stations they were recently replaced by automatic devices (e.g., in Finland). Snow gauges are employed to measure the SWE of new snow in Albania, Bosnia and Herzegovina, Bulgaria, Czech Republic, Finland, France, Hungary, Iceland, Ireland, Italy, Poland, Republic of Macedonia, Romania, Russia, Slovakia, Spain, and Ukraine. SWE is measured with the Snowpack Analyzer in Bulgaria, Czech Republic, Italy, Poland, Slovakia, Sweden, Turkey, and the U.K.; and with the snow pillow in Bulgaria, Czech Republic, Italy, Norway, Poland, Spain, Sweden, Switzerland, and Turkey. Ground penetrating radars are employed in Austria, France, Iceland, Norway, Poland, Spain, Sweden, and Switzerland. Gamma and cosmic ray sensors are utilized only in Finland, France, Iceland, Norway, and Spain, and the acoustic sensor are used in Germany. Automatic instruments to measure snow presence (such as camera or web-cam, snow depth sensors, and infrared sensors) are utilized by all countries, except Bosnia and Herzegovina, Bulgaria, Croatia, Lithuania, Portugal, Republic of Macedonia, Romania, Serbia, and Ukraine.

**Figure 7:**
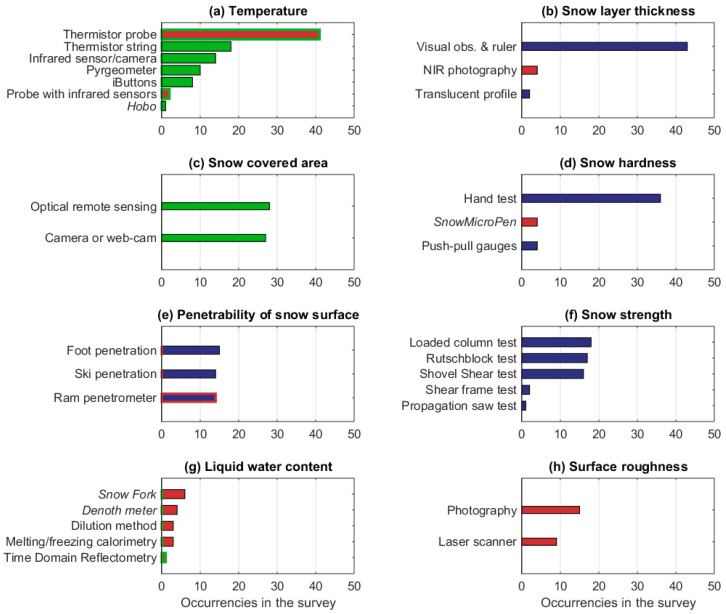
As in Figure 6, but for snow temperature (**a**), snow layer thickness (**b**), snow cover area (**c**), snow hardness (**d**), penetrability of the snow surface (**e**), snow strength (**f**), liquid water content (**g**), and surface roughness (**h**). Different colours denote manual instrument without electronics (blue), manual instrument with electronics (red), manual instrument with or without electronics depending on the version of the instrument (blue with red contour), manual instrument with electronic or fully automatic depending on the version of the instrument (red with green contour), and fully automatic instrument (green).

The bulk density of the snowpack cannot be directly measured with automatic methods, but it can be indirectly inferred from the collocated automatic measurements of SWE (e.g., using a scale, a snow pillow, a neutron probe, or a Snowpack Analyser) and HS (e.g., using ultrasonic or laser depth sensors), or from the snow penetration resistance measured with the *SnowMicroPen*. Snow density can also be measured with the *Snow Fork* and the *Denoth Meter*, but these instruments were listed in the questionnaire only among the possible measurement methods for liquid water content (Figure 7), as this is the quantity for which they are mostly designed. 

The instruments that are used to measure the other macrophysical properties are shown in Figure 7. These properties are much less frequently measured by the respondents than those listed in Figure 6 (see Figure 5). Snow temperature (the vertical temperature profile of the snowpack or just the temperature of the surface layer) is measured by 46% of the respondents, 74% of whom do manual measurements of the snow temperature profile using a thermistor probe. However, several automatic techniques are also applied; the vertical thermistor strings that are used to measure the vertical temperature profile of the snowpack had the highest occurrence after thermistor probe, followed by infrared sensors and pyrgeometers (applied to measure the surface temperature), and iButtons and probes with infrared sensors (applied to measure the vertical temperature profile of the snowpack). The latter instruments are based on a more advanced technology than the previous and are used by two respondents only. In the literature, also other thermometers such as thermocouples (handheld or in fixed arrays) have been applied to measure snow temperature (e.g., [15,26]) but they were neither listed among the possible choices in the questionnaire nor reported by the respondents in the option ‘other’, as they are evidently not commonly used by European institutions.

Although snow covered area is a fundamental property comparable to those shown in Figure 6, it is mostly derived from satellite observations, and hence, it is measured in the field only by 32% of the respondents. Excluding temperature and snow cover area, the other properties in Figure 7 are only manually measured. Snow hardness, penetrability of the snow surface, and snow strength (or shear resistance) are applied in the assessment of avalanche risk, therefore they are mostly measured by the operational institutions or research centres that are responsible for avalanche warnings and/or research. Among the listed tests to assess the snow strength (Figure 7f), only the shear frame really measures the snow strength (see Table A6). The other methods provided an estimation of the stability of the snowpack, and, hence, a qualitative evaluation of its strength. Measurements of liquid water content in snow, which are used for forecasting wet snow avalanches and melt-water runoff [27], and for snow mass and energy budget estimations, are quite challenging, and are performed by 14% of the respondents only. For these measurements, the Snow Fork is used in Finland, Lithuania, and Slovakia; the Denoth meter in Austria, Italy, and Switzerland; the dilution method in Croatia, Russia, and Spain; the melting/freezing calorimetry in France, Iceland, and Slovenia; while the time domain reflectometry is used in Romania. Surface roughness measurements are made to better interpret the optical and microwave signature of the snow, and are performed by a very limited group of scientists (15 respondents, corresponding to 13% of the total). To estimate the surface roughness, photography is applied in Finland, France, Italy, Poland, Russia, Spain, and the U.K., while laser scanner is applied in Finland, France, Italy, and Spain. Another measured snow macrophysical property that was not included in Figure 6 and Figure 7 is the snow thermal conductivity that is measured by 10 participants using heat flux plate (Ireland, Poland, and Switzerland), transient needle probe (France and Switzerland), or derived from micro-computed tomography of casted snow samples (France and Switzerland) or thermistor strings (Finland, Iceland, Poland, and the U.K.). Also not shown in Figure 6 and Figure 7 are meltwater runoff, measured by eight respondents with the lysimeter (in Czech Republic, France, Latvia, Norway, Russia, Slovakia, Spain, Switzerland, and Turkey), and the snow aerodynamic roughness (roughness length) that is obtained by five respondents from the vertical profile of the wind speed (this quantity, however, is mostly applied in meteorological applications, and therefore it is mostly measured by a community different from the one that was targeted by this survey).

#### 3.3.2. Snow Microphysical Properties

**Figure 8:**
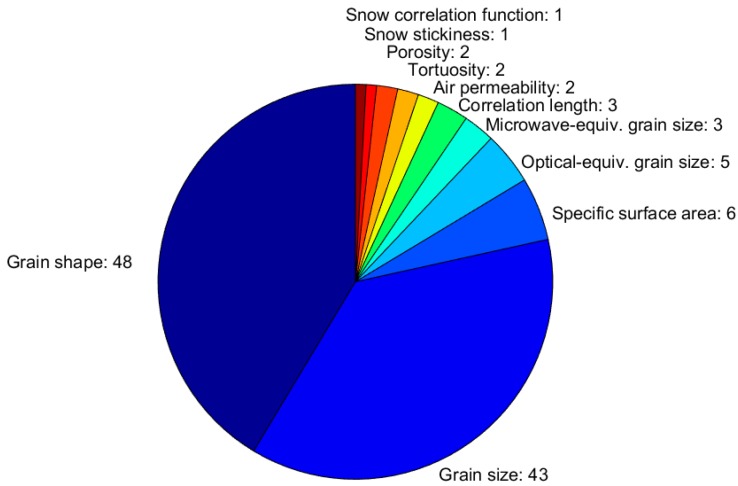
Measured snow microphysical properties. The width of each slice corresponds to the fraction each snow microphysical property has, relative to the total number of reported microphysical properties (i.e., the sum of the numbers given in the figure).

**Figure 9:**
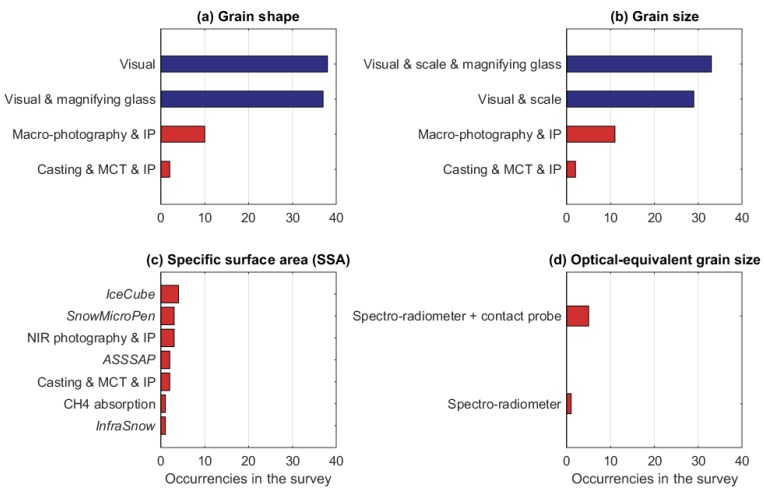
Occurrences of the applied instruments to measure snow microphysical properties, such as grain shape (**a**), grain size (**b**), specific surface area (SSA) (**c**), and thermal conductivity (**d**). The abbreviations IP and MCT stand for image processing and micro-computed tomography, respectively. Different colors denote manual instrument without electronics (blue) and manual instrument with electronics (red).

Of the respondents, 51 (41% of the total) measured snow microphysical properties, generally for research purposes. The number of respondents measuring each microphysical property, and the fraction of each measured property over the total amount of measured microphysical property is given in Figure 8. It is worth noting that only two parameters, namely snow grain shape and size, are observed by most of the respondents (48 and 43, respectively, corresponding to 94% and 84% of the respondents measuring microphysical properties), the majority of whom applies traditional visual methods (Figure 9). Others parameters are measured by few groups only. Indeed, Figure 9 reveales that only a few research groups apply macro-photography with image processing (Belgium, Finland, France, Ireland, Italy, Norway, Poland, Russia, and Switzerland) or near-infrared (NIR) photography and image processing (Italy, Switzerland, and the U.K.), and even fewer use snow sample casting, micro-computed tomography, and image processing—a sophisticated method that requires specialized laboratories (only present at the Centre d’Etudes de la Neige, Meteo-France/CNRS, France, and at the WSL Institute for Snow and Avalanche Research, Switzerland). The snow specific surface area (SSA) is measured only by six respondents (from Finland, France, Italy, Switzerland, and the U.K.), each of them generally applying several techniques that were developed only recently. This is the case for the *IceCube* that was developed in France [11] and was utilized in Finland, France, Switzerland, and the U.K.; the *SnowMicroPen* was developed in Switzerland [13] and was applied in France, Switzerland, and the U.K.; the NIR-photography method was developed in Switzerland [28] and is applied in Italy (Insubria University), Switzerland (WSL Institute for Snow and Avalanche Research), and the U.K. (Northumbria University); the *ASSSAP* [12] was developed and is applied in France; and the *InfraSnow* [29] was developed and is applied in Switzerland. These new techniques are much more practical and ‘portable’ than the more traditional methods that are based on gas absorption or micro-computed tomography, which require specialized laboratories. The methods that are applied to measure SSA are used to derive also other microstructural properties; the *SnowMicroPen* is applied to estimate the snow correlation length and density, while the micro-computed tomography of casted samples is applied to derive snow tortuosity, porosity, stickiness, anisotropy, and air permeability.

The optical- and microwave-equivalent grain size are measured by five and three respondents, respectively (optical-equivalent grain size from Finland, France, Romania, and Switzerland, and the microwave-equivalent grain size from Finland, France, and the U.K.). 

In the questionnaire, only the spectro-radiometers were listed among the instruments that are available to derive the optically equivalent grain size (applying the radiometric measurements as the input to the inverted radiative transfer models). However, the optically-equivalent grain size is related to SSA (see Table A2), hence, in practice, all of the SSA measurements can be converted to the optical equivalent grain size and vice versa. Moreover, an estimation of the optical equivalent grain size can also be obtained from the visual observations and macro-photography of snow crystals (e.g., [30,31]).

#### 3.3.3. Snow Electromagnetic Properties

**Figure 10:**
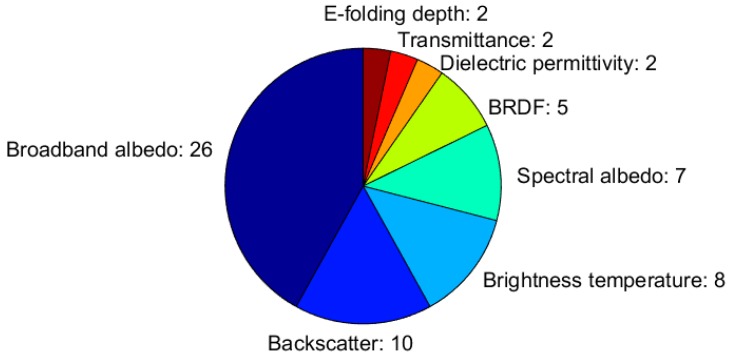
Measured snow electromagnetic properties. The width of each slice corresponds to the fraction each snow electromagnetic property has, relative to the total number of reported electromagnetic properties (i.e., the sum of the numbers given in the figure).

Figure 10 illustrates how frequently each electromagnetic property is measured among the respondents, with respect to the total number of measured electromagnetic properties. Of the respondents, 33, which corresponded to 26% of the total, measure snow electromagnetic properties, and the majority of them (26 respondents) measure the snow broadband albedo (Figure 10), mostly applying pyranometers (in 22 of the twenty-six responses). These numbers seemed small, in view of the high relevance of snow albedo for surface energy budget and water runoff calculation, and for the strong feedbacks that its variability has on climate. One explanation is that, although the pyranometers are fully automatic, they need daily manual cleaning and have demanding requirements in terms of instrument calibration/characterization (re-calibration is needed every year and the deviation from the ideal angular response need to be measured) and installation (the holding structure should be designed to minimize shadows and reduction of diffuse illumination, the target surface should be flat, and the field of view should be free from obstacles and from reflecting tilted surfaces) to ensure the correct interpretation and good accuracy of the measurements [32,33]. 

As for the other snow properties, most of the respondents measure more than one electromagnetic parameter. For instance, among the respondents who apply spectro-radiometers to measure spectral albedo (from Finland, France, Norway, and Switzerland), four derive the broadband albedo from the same instrument/dataset. In the microwave spectral region, the snow backscatter and snow brightness temperature are measured through ground-pointing radars and microwave radiometers by ten and eight respondents, respectively; only the backscattering coefficient is measured by the University of Pavia (Italy), the Norwegian Water Resource and Energy Directorate (Norway), the Swedish Meteorological and Hydrological Institute (Sweden), and University of Zurich (Switzerland), and only the brightness temperature is measured by the Grenoble-Alps University (France), the University of Oslo (Norway), the Middle East Technical University (Turkey), and Met Office (the U.K.), while the both of them are measured at the Finnish Meteorological Institute (Finland); Centre d’etudes de la neige, Meteo-France/CNRS (France); University College of Dublin (Ireland); and Northumbria University (the U.K.). From the measurements of the backscattering coefficient and brightness temperature, and by using advanced microwave models and inversion techniques, the SWE could be estimated and, potentially, information on liquid water content could be extracted. More specialized measurements, which require experimental instruments that are not commercially available, include the bidirectional reflectance distribution function (BRDF), the snow e-folding depth, and the snow optical transmittance. BRDF is measured at the Centre d’etudes de la neige, Meteo-France/CNRS (France), the Regional Environmental Protection Agency (ARPA) of Lombardia region (Italy), the University of Oslo (Norway), and the University of Zurich (Switzerland) mostly using gonio-spectro-radiometers (but in one case also applying a camera on board an Unmanned Aerial Vehicle and Lambertian-reflecting targets at the snow surface), while snow e-folding depth and optical transmittance are measured in France with fibre-optic probes. Snow dielectric permittivity can be directly measured with several instruments, which generally apply the measurements to automatically derive other snow bulk properties (see Table A3). However, a few respondents (from Finnish Meteorological Institute, Finland, and Northumbria University, the U.K.) measured the dielectric permittivity as the targeted snow property (later applied to calculate snow macrophysical properties).

#### 3.3.4. Precipitating and Suspended Snow

**Figure 11:**
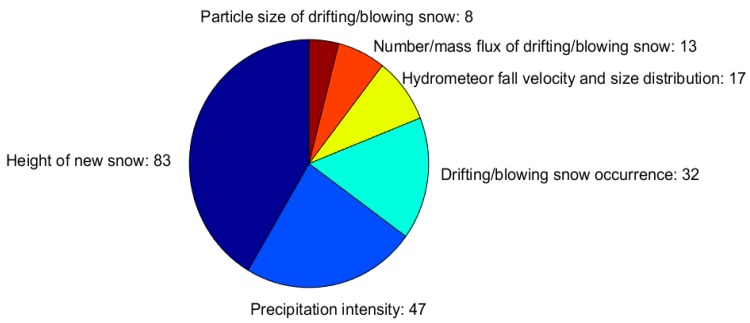
Measured precipitating and suspended snow properties. The width of each slice corresponds to the fraction each precipitating and suspended snow property has, relative to the total number of reported precipitating and suspended snow properties (i.e., the sum of the numbers given in the figure).

Of the respondents, 93 (74% of the total) measure one of the measurable properties characterizing the precipitating and suspended snow. This group of measurements includes properties, such as the height of new snow (HN, also called depth of snowfall) and precipitation intensity, which are used by operational monitoring services, and others such as the fall velocity of hydrometeors and their particle size distribution, the occurrence of drifting/blowing snow, the number flux, and particle size distribution of drifting/blowing snow, which are mainly utilized in research applications. The first properties are therefore much more frequently measured than the second ones (Figure 11).

**Figure 12:**
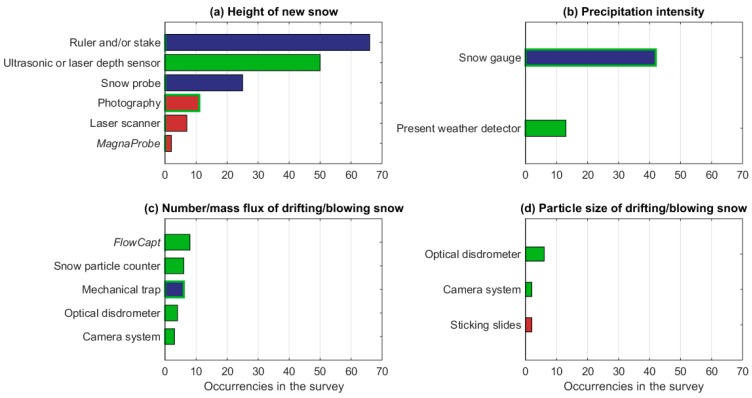
Occurrences of the applied instruments to measure the precipitating and suspended snow properties, such as the height of new snow (**a**), precipitation intensity (**b**), number/mass flux of drifting/blowing snow (**c**), and particle size of drifting/blowing snow (**d**). Different colors denote manual instrument without electronics (blue), manual instrument with electronics (red), manual instrument without electronic or fully automatic depending on the version of the instrument (blue with green contour), manual instrument with electronic or fully automatic depending on the version of the instrument (red with green contour), and fully automatic instrument (green).

Most of the respondents measure the HN (83 respondents, corresponding to 89% of those measuring precipitating and suspended snow properties), with a variety of instruments (Figure 12a), the most popular ones being rulers/stakes and ultrasonic/laser depth sensors (used by 66 and 50 respondents, respectively). To measure the HN, the recommended practice is to cover the old snow surface with a wooden or plastic board after the last measurement, as a marker for the top layer (Table A4). According to the World Meteorological Organization (WMO) guideline, “if there is a layer of old snow, it would be incorrect to calculate the depth of a snowfall from the difference between two consecutive measurements of the total depth of the fresh and old snow, because of the continuous settling of the old snow” [34]. In our questionnaire, we did not ask to specify if the reference board above the old snow layer is applied or not. In Finland, for instance, such a board is not used, which imply that the HN measurements are not reported by the environmental and meteorological snow monitoring agencies. In Germany, on the contrary, the reference board is used for both manual and automatic HN measurements, and an optimized board has recently been designed and applied to the observational network [35]. As in the case of HS, the measurement of HN is mostly done using manual instruments, the only automatic devices being ultrasonic/laser depth sensors and digital cameras that are set to collect lapse-rate photos. 

The precipitation intensity is recorded by 47 respondents, mostly using a snow gauge (Figure 12b). This snowfall property is generally measured automatically; however, traditional manual gauges are still utilized. The hydrometeor fall velocity and size distribution are measured with optical disdrometers by eight respondents. For research applications, disdrometers are also used to provide an estimate of the particle size and type. Present Weather Detectors (PWDs) can observe the precipitation intensity and type, as well as the visibility. Optical probes, which are developed for airborne use, are also, in some cases, used on the ground for measuring particle size precipitation. Of the 39 respondents measuring drifting/blowing snow properties, all but one also measure the precipitating snow properties, and 32 do visual measurements of the occurrence of drifting/blowing snow. The number or mass flux of drifting snow is measured by thirteen participants applying a large variety of techniques that are all automatic (Figure 12c), except the traditional manual version of the mechanical trap (See Table A6 in Appendix B). While the *FlowCapt* and the mechanical trap measure the mass fluxes of snow particles, the Snow particle counter (SPD), the optical disdrometer, and the camera system detect the number fluxes of snow particles. The particle size of the drifting/blowing snow is measured by ten respondents, eight of them applying automatic devices. Only two respondents (from Russia and Slovenia) use sticking slides, which need to be manually removed and photographed (see Table A6 in Appendix B).

#### 3.3.5. Snow Composition

**Figure 13:**
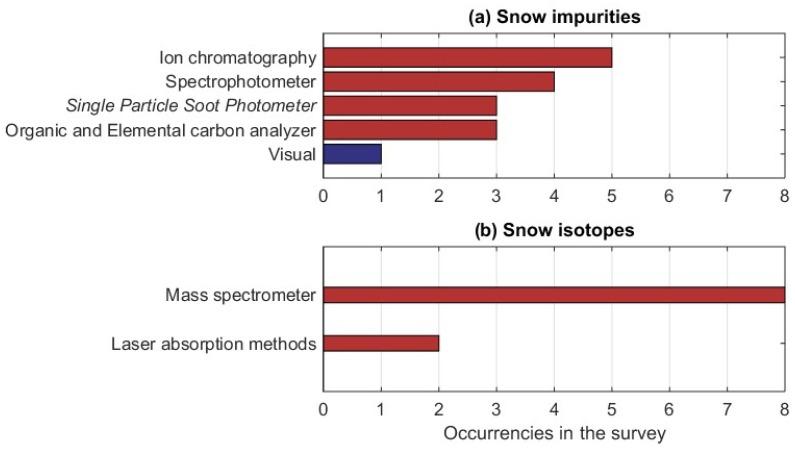
Occurrences of the applied instruments to measure snow impurities (**a**) and snow isotopes (**b**). Different colors denote manual instrument without electronics (blue) and manual instrument with electronics (red).

The snow composition properties are measured by only 16 respondents (corresponding to 13% of the total). The snow impurities are measured by ten respondents (from Austria, Finland, France, Italy, Poland, Switzerland, and Ukraine), with ion chromatography being the most applied measurement method (Figure 13a). A visual, qualitative estimation of the snow impurities was carried out only in Ukraine. All of the other methods rely on the collection of snow samples that are then analysed later, with equipment that can only operate inside laboratories. Thus, these measurement methods are all classified as ‘manual with electronics’ in Figure 13a (see the description in Table A6 of Appendix B). Different instruments are applied to measure different impurities; ion chromatography is used for dust, SP2 for soot or small black carbon (BC) particles, and the organic and elemental carbon (OC/EC) analyzer for a wider spectrum of organic components. Water and heavy metal isotopes measurements also require the collection of snow samples and their successive laboratory analysis. They are performed by nine respondents (from Austria, Iceland, Italy, Poland, Slovakia, Slovenia, and Spain), most of whom apply a mass spectrometer (Figure 13b).

### 3.4. Use of Measurement Protocols

Of the respondents, 75 (60% of the total) use a written protocol for some of the measured snow properties, and 22 of them (all but one from the operational oriented institutions) use it for all of the measured properties (Table 1). For all of the well-established measurement techniques, a written protocol exists, to ensure the homogeneity and inter-comparability of the measurements and to reduce the error sources. This is especially important for those properties that are measured for the operational applications. Nevertheless, among the 71 respondents from operational oriented institutions, 18 do not apply any written protocol. Different environmental conditions, however, may require the adaptation of the protocol to the local needs. In case of the devices that are used for research applications, there may not be any written measurement protocol, especially when the instruments are developed by the research groups and are not commercially available. 

## 4. Summary and Discussion

The survey was answered by most of the European national weather agencies and by several national institutions that are responsible for water/environmental management and avalanche forecasts, and by numerous universities and research institutes. However, not all of the European research and operational institutions that are involved in the snow measurements responded to the survey. This is mainly because of the lack of centralized and institutionalized coordination of the snow measurement activities at national level. Moreover, some of the participants provided answers that cover the activities that are carried out in the whole institution they represented, while others just described the measurements that are done by themselves or their group. Indeed, there were no clear instructions on how comprehensive the answers should have been, and the position of the respondents in their own institution varied a lot, from technician and PhD student to professor, and to the head of weather service or of measurement division. Despite these limitations, the survey managed to capture the state-of-the-art of the European in-situ snow operational monitoring and research-driven snow measurements. 

Strictly speaking, the percentages of respondents that measure each of the snow properties do not directly indicate how diffusely or frequently those properties are measured, but rather illustrate the measurement capacities of the respondents (what is measured and how it is measured). However, the received answers provided a quite strong indication on what are the most commonly measured properties and applied techniques. The respondents from the operational oriented institutions were more numerous than the respondents from the research only institutions, but the research objectives were, in most cases, shared by both, thus research was the dominant measurement purpose that was marked in the survey. On the other hand, snow research is largely driven by the needs of the operational services, therefore, the most widely measured snow properties are the few ones that are utilized by the operational monitoring services. These include snow macrophysical properties, such as snow presence, HS, SWE, and snow density, which are measured by 93% of the respondents, and the snow precipitation quantified as the height of new snow, which is measured by 66% of the respondents. A large variety of manual and automatic instruments are applied in order to collect these properties, with the manual instruments being the most diffuse and with the longest application history (e.g., [36]). The manual measurements of the HS are mostly reported only in presence of snow, without distinguishing the cases of zero HS and of missing observations. This reporting practice leads to a bias in the HS data records, which is an issue for the data assimilation applications. This issue was addressed by the WMO 69th Executive council in May 2017, to improve operational exchange of snow data and reporting practices.

Our analysis showed that most of the respondents that apply the manual method also apply automatic techniques. Important drivers for the automatization of the snow macrophysical properties are the snow and avalanches services in mountain areas. In order to be able to provide the public with timely information about the snow situation and the avalanche hazard, automatic stations with a variety of reliable meteorological and nivological sensors are needed. These data are often used to drive snow models, which provide even more information in high temporal resolution. Nevertheless, manual observation of, for example, the height of new snow, amount of wind transported snow, or possible weak layers, are still an important part of a high quality snow and avalanche information service. Such in-situ snow observational networks need to achieve a high level of automatization to ensure the harmonization, the distribution across institutions, and the long term sustainability of the measurements, with direct manual measurements ideally performed only for the calibration and validation of the automatic methods. The results of this survey demonstrated that many European institutions are engaged in an ongoing effort toward this goal.

Some new automatic technologies that are developed and exploited by the European community have been transferred or have the potential to be transferred from the research to the operational domain, for instance the Snowpack Analyzer [37] and GPS reflectometry [38,39,40] to derive the snow bulk properties, and the web cam network to retrieve the snow cover fraction [41]. While some of the new automatic methods (e.g., those based on cameras) have a low price and, therefore, can possibly reach a more widespread usage, others that are based on expensive technology (e.g., Neutron probe, GPR Radar, and Gamma and cosmic ray sensors) cannot be adopted by a large community. An increase in the measurements of the snow bulk properties across institutions is envisaged also in view of the on-going efforts made by ECMWF and WMO to define the SWE data exchange protocol for operational NWP applications. Indeed, currently there is not an exchange protocol for the SWE measurements among the operational networks, thus SWE is not used in the NWP data assimilation systems. Present-day operational NWP systems only rely on HS measurements, which are exchanged on the operational networks supported by WMO.

In addition to the data exchange protocols, written measurement protocols are recommended, especially for the operationally relevant snow properties, to ensure the continuity of the measurements, the transfer of knowledge among different operators of the instruments, and the cooperation among the scientific community that use or develop similar instruments. In principle, the measurement protocols should be internationally agreed and shared, to ensure the comparability of the data and to facilitate their transfer. In practice, protocols need to be tailored to the specific measurement sites, because the measurement issues are dependent on the sites and their meteorological conditions (e.g., average wind speed and amount of snowfall), as observed in the Solid Precipitation Intercomparison Experiment (SPICE) that was organized by WMO in 2012–2015 [42].

The survey also clearly illustrated that the automatization of the instruments is dependent on the measured variable. Snow microphysical and composition variables, for instance, cannot yet be easily measured by fully automatic instruments (Figure 9 and Figure 13). This is a major limitation for the operational services, as they would need to put significant effort into the collection of data if it requires personnel for field work. In practice, the consequence is that snow microphysical and composition variables will not be widely monitored in Europe until a higher degree of automatization is reached. However, the monitoring of snow microphysical properties would be highly beneficial for many operational services. The availability of measurements of snow microstructure (optical- and microwave-equivalent grain size, SSA, correlation length) would support the development of assimilation schemes that use satellite-based radiances as input. The model analysis field could be produced using satellite-based snow microstructure, snow extent, albedo, SWE, and HS, and by applying in-situ observations of HS and snow microstructure to constrain the satellite data. The benefits of this physically consistent assimilation approach were recently demonstrated in the case of a snowpack model that was used for avalanche risk forecasting [43], but they most likely exist also in numerical weather prediction and hydrological models. Microstructural properties, such as snow stickiness, porosity, and tortuosity, which determine the elasticity of the snow [44], are derived from the extremely laborious X-ray tomography, which can be performed in a couple of European laboratories only. More observations of these quantities would be needed to develop models that describe them, and that could have many operational applications (warning on road slipperiness, forecasting snow surface conditions for winter sports and snow loads on trees, buildings, and wind turbines, etc.).

The in-situ measurements of the most common snow electromagnetic properties (broadband albedo, backscatter, and brightness temperature) are done with fully automatic instruments (see Table A6). In the microwave spectral region, the signal received from the radars and radiometers is used to derive the snow extent, SWE, and snow melting/freezing state. These measurements are mainly applied for research purposes, such as the ground truth for satellite observations, and the development/validation of snow retrieval algorithms and of models for the snow-electromagnetic interaction. The in-situ monitoring of the snow extent, SWE, and snow melting/freezing state is delegated to the cheaper and more practical instruments shown in Figure 6 and discussed in the paragraphs above. Although the snow broadband albedo is a crucial variable for the climate, weather forecasting, and snow mass and water runoff estimation, it is measured by 21% of the respondents only. A larger number of broadband albedo measurements is probably hampered by the demanding maintenance and installation requirements of the pyranometers (as discussed in Section 3.3.3). A clearer measurement protocol would possibly help in increasing the number of the in-situ broadband albedo measurements, which are now mainly used for the development and validation of models that simulate the radiative transfer in snow, snow albedo schemes, and snow retrieval algorithms, and for the validation of snow satellite products. 

Among the snow properties related to precipitating and suspended snow, the HN and precipitation intensity are the only ones applied as input in numerical weather prediction, hydrological models, and snow models used for avalanche risk forecast (e.g., [45,46]). The microphysical properties of precipitating and suspended snow (hydrometeor fall velocity and size distribution, particle size and number flux of drifting snow) are mainly measured with automatic devices, mostly for research purposes, such as the validation of polarization radar measurements and the assessment of snow transport in avalanche terrain [47,48] and polar regions [49,50,51,52]. These measurements would also be very important input parameters for the physical snow models that simulate the snow albedo, to constrain the optical equivalent grain size and albedo of fresh snow. From the operational perspective, observations of drifting snow would be extremely beneficial in the management of traffic along roads, rails, and runaways in locations dominated by strong winds, and to assess the risks related to the transport of snow over infrastructures and on avalanche-prone terrains. The transfer of microphysical measurements of precipitating and drifting snow from research institutions to operational services would require proper instrument calibration (see, for example, [53]) and the automatization and standardization of the software applied to interpret the signals acquired by the automatic optical and acoustic sensors (disdrometer, *FlowCapt*, and snow particle counter).

## 5. Conclusions

The survey on the practices and purposes of the European in-situ snow measurements, carried out in the framework of Harmosnow, enabled the collection of a compilation of the state-of-the-art of in-situ snow operational and research-based snow measurements conducted by the European countries, in a time when new technological solutions are tested and developed. As such, it constitutes the basis for the upgrade and harmonization of the in-situ observation methods, which is one of the main goals of Harmosnow, and for the enlargement of the communities that applied automatized measurements for operational monitoring and frontier techniques for research purposes. The mapping of snow monitoring in Europe obtained through this survey is the needed pre-requisite for the identification of observational gaps and the planning of future long-term snow measurement strategies. Although the snow operational monitoring largely relies on satellite-based observations, in-situ measurements are an essential component of it, as both satellite and ground-based observations are applied in the snow assimilation schemes (e.g., [54,55,56]). Hence, the enhancement and harmonization of the in-situ monitoring will improve the description and the assimilation of the snow cover information into hydrological, land surface, meteorological, and climate models. This is critical for addressing the impact of snow on various phenomena, to predict local snow water resources, and to warn about snow-related natural hazards. The results of this compilation are therefore discussed, also, from the perspective of the need of enhancing the efficiency and coverage of the in-situ observational network by applying automatic measurement methods. The results showed that (1) some of the automatic instruments that are used to measure bulk snow properties have good potential for being applied to monitoring large and remote areas (GPS reflectometry, photography, etc.); (2) some of the snow properties that are presently measured only manually for research applications would be greatly beneficial for operational services (snow stickiness, SSA or snow optical/microwave equivalent grain size, and snow correlation length); (3) the snow drift properties (number flux and particle size distribution) that are automatically measured and are mostly used in the research applications would already be (or close to be) mature to be applied operationally to provide specific services (e.g., information on snow drift over roads, runaways, and rails). This leads to few final recommendations, namely: automatic instrumentation should be adopted, especially when cheap and practical solutions are available, and the properties that are presently measured for research applications would be needed and should be acquired also by operational networks, particularly when they are collected with automatic instruments. Moreover, the development and the increase in the use of internationally agreed measurement protocols for each of the applied measurement techniques are strongly encouraged, as their application will enhance the harmonization of the measurements. This is in line with the recent Global Cryosphere Watch Initiative for improving in-situ HS and SWE observations [57].

These results are relevant for the planning of snow observation infrastructures and monitoring programs, as well as for the scientific community—the specialists in specific applications who are familiar with a certain set of snow properties, will generally benefit from knowing the measurement practices that are applied by different communities and in different environments. The measurement practices that are developed on the basis of the knowledge acquired in local institutions and for targeting specific environments will get visibility among a wider audience, eventually broadening their applicability also in other contexts. Moreover, snow properties that are measured for certain purposes may be very relevant for other applications too. Indeed, this work is in line with the recommendation of sharing and comparing techniques between the snow monitoring teams provided by the recent review on the changing Arctic snow cover [58], which highlighted the need for an interdisciplinary approach to resolve the current snow measurement and modelling limitations.

The effort spent to send the survey to as many institutions as possible, and the difficulties encountered in order to identify the key responsible persons for snow measurements, evidenced that a much better intra-national collaboration and knowledge-exchange of snow measurement activities is necessary in Europe. The current fragmentation and peculiarities represent a major bottle neck for the necessary harmonization of snow measurements practices in Europe.

## Figures and Tables

**Table 1:** Optional answers available to the question “Did you apply a written protocol (published or unpublished, written in any language) when measuring the snow parameters?”, and the number of given answers per option.

N° of Answers	Answer
22	**Yes**, for all of the measured parameters I applied a written protocol, which describes both the use of the instrumentation and its set up in various environmental conditions.
53	**Yes**, for some of the measured parameters I applied a written protocol, which describes both the use of the instrumentation and its set up in various environmental conditions. For some parameters I only followed the general instructions of the instrument and applied measurement strategy based on my own experience or on the oral teaching of senior scientists/operators.
37	**No**, I only followed the general instructions of the instrument and applied measurement strategy based on my own experience or on the oral teaching of senior scientists/operators.
8	**No**, I do not use any written protocol, and I apply the instrument’s instructions using the instrument as I learned to, by myself.
5	**Other**

## Appendix A. List of the Measurable Snow Properties

The measurable snow properties are divided into five groups, according to the associated snow properties, as follows: snow macrophysical properties (Table A1), snow microphysical properties (Table A2), snow electromagnetic properties (Table A3), precipitating and suspended snow (Table A4), and snow composition (Table A5).

**Table A1:** List of measurable snow macrophysical properties: name of the properties (first column), definition (second column), and applicable instruments.

Parameter	Definition	Instruments
**Runoff**	Melted water that flows over land as surface water	- Lysimeter
**Snow covered area, or snow cover extent (SCE)**	Snow covered area is defined as the areal extent of the snow-covered ground, usually expressed as a fraction (%) of the total area investigated. The latter must be defined (e.g., observation site, catchment, district, country, continent). Unless otherwise specified, only seasonal snow cover is considered. Hence, on glaciers and névés, ground refers either to glacier ice or to an old firn surface [14].	- Optical remote sensing - Camera or web-cam
**Snow density**	Density, that is, mass per unit volume (kg m^−3^), is normally determined by weighing snow of a known volume. Sometimes total and dry snow densities are measured separately. Total snow density encompasses all of the constituents of snow (ice, liquid water, and air), while the dry snow density refers to the ice matrix and air only [14].	- Snow tube - Snow cutter + scale - Scale + snow depth measurement - Neutron probe - *SnowMicroPen* - *Denoth Meter*
**Snow depth (HS)**	HS denotes the total height of the snowpack (i.e., the vertical distance in centimetres from base to snow surface). Unless otherwise specified, HS is related to a single location at a given time. Thus, the manual HS measurements are often made with one or more fixed snow stakes. On the other hand, portable HS probes allow for measurements along snow courses and transects. The slope-perpendicular equivalent of the HS is the total thickness of the snowpack, denoted by DS [14].	- Stakes - Rulers - Snow probe - *MagnaProbe* - Ultrasonic and laser depth sensors - Time-lapse photography - Terrestrial and air-borne Laser Scanner - Unmanned Aerial Vehicle + Photogrammetry - Ground Penetrating Radar (assuming a constant average density)
**Snow hardness**	Hardness is the resistance to penetration of an object into the snow. Hardness measurements produce a relative information value that depends on the instrument. Therefore, the device has to be specified [14].	- Hand test - Push-pull gauges - *SnowMicroPen* - *Rammsonde*
**Snow layer thickness (stratigraphy)**	The snow layer thickness (measured in centimetres or fractions thereof) is an essential parameter when characterizing the current state of a snowpack. Layer thickness is usually measured vertically. If the measurement is taken perpendicular (i.e., slope normal), the layer thickness should be denoted by L_p_ [14]. The definition of a layer is somewhat arbitrary and observer dependent.	- Visual observations + ruler - Near Infrared (NIR) photography - Translucent profile
**Snow liquid water content (LWC)**	Liquid water content is defined as the amount of water within the snow that is in the liquid phase. This parameter is synonymous with the free-water content of a snow sample. Liquid water in snow originates from either melt, rain, or a combination of the two. Measurements of liquid water content or wetness are expressed as either a volume (LWC_V_) or mass (LWC_m_) fraction. Both can be reported as a percent (%), and always require a separate measurement of density [14].	- Melting/freezing calorimetry - Alcohol calorimetry - Dilution method - Time domain Reflectometry (TDR) - *Snow Fork* - *Denoth Meter*
**Snow (surface) penetrability**	Penetrability is the depth that an object penetrates into the snow from the surface. It can be used as a rough measure of the amount of snow available for transport by aeolian processes, or the ability of a snowpack to support a certain load. The depth of penetration of some suitable object (such as a *Rammsonde* element, a foot, or a ski) is measured in centimetres [14].	- *Rammsonde* - Foot penetration - Ski penetration
**Presence of Snow**	A binary observation of the presence of snow cover at the measurement location, usually based on a snow cover fraction threshold of 50%.	- Visual - Camera or web-cam - Snow depth sensor - Thermometer - Infrared sensor
**Snow (surface) roughness**	It refers to the roughness of a snow surface caused by the precipitation, or the wind, as well as by uneven sublimation or melt. It does not refer to the roughness as a result of the snow microstructure.	- Laser scanner - Photography
**Snow roughness length (aerodynamic roughness)**	It is used in some vertical wind profile equations that model the horizontal mean wind speed near the snow covered ground. In the log wind profile, it is equivalent to the height at which the wind speed theoretically becomes zero. Drag coefficient is an alternative measure of the same quantity.	- Derived from wind profile or eddy-covariance
**Snow strength/shear resistance**	Snow strength can be regarded as the maximum or failure stress on a stress–strain curve. It is the maximum stress snow can withstand without failing or fracturing. Snow strength depends on the stress state (*σ*: compressive or tensile; *τ*: shear) in pascals and the strain, *ε*, which is dimensionless, as well as on their rates in pascals per second and per second, respectively. Snow strength depends also on microstructure and on the homogeneity of the sample. To make measurements meaningful, all of these properties must be considered. Moreover, failure types such as ductile or brittle fracture or maximum stress at low strain rates must be given [14]. Various tests have been developed (listed on the right) to assess the stability of the snowpack, and, thus, provide a qualitative evaluation of its strength. Only the shear frame provides a real measurement of the strength.	- Shovel Shear test - Loaded column test - Trapezoidal Tensile test - Flat jack Shear test - Shear frame test - Rutschblock test - Propagation saw test
**Snow temperature (Ts)**	T is defined as the temperature of the snow. It can be taken at the snow surface and/or at various depths of the snowpack.	- Handheld thermometer - Fixed thermistor string - Pyrgeometer (only surface) - Infrared sensor (or camera) above the surface (only surface) - Probe with infrared sensors - iButtons - *HOBO*
**Snow thermal conductivity**	Property of snow that controls the heat conduction in the snow	- Heat flux plate - Transient needle probe - Thermistor string + modelled or measured heat flux - Micro-computed tomography + image processing
**Water equivalent of the snow (snow water equivalent [SWE])**	The water equivalent of the snow cover is the vertical depth of water that would be obtained if the snow cover melted completely, which equates to the snow-cover mass per unit area. It can represent the snow cover over a given region or a confined snow sample over the corresponding area. The snow water equivalent is the product of the snow height in meters and the vertically-integrated density in kilograms per cubic meter. It is typically expressed in millimeters of water equivalent, which is equivalent to kilograms per square meter or liters per square meter, thus referring to the unit surface area of the considered snow sample [14].	- Snow tube - Snow cutters + scale - Snow pillow - Scale - Snow gauge - Snowpack analyzer - Gamma and cosmic rays sensors - Ground penetrating radar - Acoustic sensor - Global positioning system (GPS) - Reflectometry

**Table A2:** List of measurable snow microphysical properties: name of the properties (first column), definition (second column), and existing instruments.

Parameter	Definition	Instruments
**Air permeability of snow**	The air permeability of snow is the property of snow that controls the ease with which a fluid, typically air or water, can move through the snow [59,60]. A direct comparison between the permeameter and numerical simulations was made by [61].	- Permeameter, or permeability apparatus (controlled air flow through a snow sample) - Casting + micro-computed tomography
**Snow (auto) correlation function**	It describes how the snow microstructures at different positions are related. More specifically, the correlation function A quantifies how microstructure variables co-vary with one another on average across space. Usually it is applied to the phase indicator function of the medium [62].	- Casting + micro-computed tomography + image processing
**Snow correlation length**	Derivative of the three-dimensional, spatial autocorrelation function A(x), with A(0) = 1, and with x being the scalar displacement [63].	- Casting + micro-computed tomography + image processing - *SnowMicroPen*
**Snow grain shape**	The main morphological classes of grain shapes are: precipitation particles (PP), machine made snow (MM), decomposing and fragmented precipitation particles (DF), rounded grains (RG), faceted crystals (FC), depth hoar (DH), surface hoar (SH), melt forms (MF), ice formations (IF). This basic classification is augmented by subclasses, where a process-oriented characterization of all of the sub-classes supplements the morphological classification. This side-by-side representation of morphological classification and physical processes should help various user groups arrive at a more reliable classification and an easier physical interpretation of their observations [14].	- Visual - Visual + magnifying glasses - Macro-photography + image processing - Casting + micro-computed tomography + image processing
**Snow grain size**	The classical grain size (E) of a snow layer is the average size of its grains. The size of a grain or particle is its greatest extension, measured in millimeters [14]. Alternatively, E can be expressed using the following terms: very fine (<0.2 mm), fine (0.2–0.5 mm), medium (0.5–1.0 mm), coarse (1.0–2.0 mm), very coarse (2.0–5.0 mm), and extreme (>5.0 mm). Some users will want to also specify the average maximum size E_max_ or even a distribution of the sizes. Note that the grain size must be regarded as a property of the snow layer and not of the grain shape or shapes.	- Visual + scale - Visual + scale + magnifying glass - Macro-photography + image processing - Casting + micro-computed tomography + image processing
**Snow microwave-equivalent grain size**	The microwave-equivalent grain size (MGS) is calculated by inverting snow microwave emission models with snow brightness temperature as an input parameter in some specific conditions, which must be specified. MGS can be related to the optical-equivalent grain size via a ‘scale factor‘ that depends on the stickiness of the snow particles.	- Microwave radiometers + snow microwave emission model
**Snow optical-equivalent grain size**	The optical-equivalent grain size (OGS) can be defined as the radius of a collection of mono-disperse modelled crystals (often spheres) with the same volume-to-surface ratio as the non-spherical snow particle [64]. In case of randomly-oriented convex snow crystals, the optical-equivalent grain size (*r_o_*) is related to SSA and, therefore, to the microstructure of snow through the following relationship: $r_{o}=3/\rho_{i}SSA$, where *ρ_i_* is the ice density.	- Contact probe of Spectro-radiometer + snow-radiative transfer model - Spectro-radiometer + snow-radiative transfer model - NIR photography - *InfraSnow* - IceCube - ASSAP - Macro-photography + image processing - Visual + scale + magnifying glass
**Snow porosity**	It is defined as the volume of the pore space divided by the total volume. Porosity is directly related to snow density.	- Casting + micro-computed tomography + image processing
**Snow specific surface area (SSA)**	SSA is defined as the total surface area of the air/ice interface, either per unit mass of a snow sample (SSA_m_) or per ice volume (SSA_V_) given in m^2^ kg^–1^ or m^2^ m^–3^, respectively. The density of ice, *ρ_i_*, simply relates SSA_m_ to SSA_V_ = ρ_i_ SSA_m_. It is related to the optical radius or effective grain size.	- IceCube - ASSSAP - Near Infrared (NIR) photography - *SnowMicroPen* - Casting + micro-computed tomography + image processing - CH_4_ absorption
**Snow stickiness**	It is a measure of the degree in which snow particles are attached to each other (by sintering and interlocking).	- Casting + micro-computed tomography+ image processing
**Snow tortuosity**	It is defined as the ratio of two distances, namely: the path between two points through either the ice or pore space and the straight line between them. The tortuosity of the ice matrix may be the primary factor determining the thermal conductivity of snow [65]. It also affects the elastic modulus and heat and mass fluxes through the pore space. Currently, tortuosity measurements require three-dimensional reconstructions of the ice/air matrix, combined with numerical simulations [66].	- Casting + micro-computed tomography + image processing

**Table A3:** List of measurable snow electromagnetic properties: name of the properties (first column), definition (second column), and existing instruments.

Parameter	Definition	Instruments
**Polarization of snow optical reflectance**	Direction of oscillation of the electrical field of an electromagnetic wave that has been reflected from the snow surface.	- Gonio-spectro-polarimeter
**Snow backscattering coefficient**	The amount of energy reflected back to the direction from which an electromagnetic pulse has been emitted, relative to the emitted amount of energy.	- Radar - Lidar
**Snow bidirectional reflectance distribution function (BRDF)**	It is defined as the ratio between the reflected radiance at a specific reflection angle and the incidence irradiance at a specific incident solar angle for each solar shortwave wavelength. In other words, it is the directional distribution of the diffuse radiation reflected by the snow surface in the solar shortwave wavelength range (350–2500 nm).	- Gonio-spectro-radiometer - Camera + white Lambertian-reflecting target
**Snow brightness temperature**	A descriptive measure of radiation in terms of the temperature of a hypothetical black-body emitting an identical amount of radiation at the same wavelength.	- Microwave radiometer
**Snow broadband albedo (α)**	It is defined as the upward broadband shortwave irradiance divided by the downward broadband shortwave irradiance. It can be computed by the integration of the spectral albedo over all of the shortwave wavelengths weighted by the actual incident spectrum.	- Pyranometers - Spectro-radiometer connected to cosine receptor or integrating sphere
**Snow directional-hemispherical reflectance**	It is defined as the integral of BRDF over a particular reflection cone.	- Spectro-radiometer
**Snow e-folding depth**	The depth of snow over which light intensity reduces by a factor 1/e.	- Fiber-optic probes
**Snow dielectric permittivity**	A measure of the resistance that is encountered when forming an electric field in the snow.	- *Snow fork* - *Denoth meter* - Time domain reflectometry - *Snowpack analyzer*
**Snow optical transmittance**	It is the property of snow to transmit light through it. It determines the portion of incoming light that is transmitted through the snow instead of being absorbed or scattered.	- Array of spectro-radiometers (two or more)
**Snow spectral albedo (α_λ_)**	It is defined as the spectral directional-hemispherical reflectance (i.e., the integral of BRDF over all of the reflection angles). In other words, the spectral albedo is the upward shortwave irradiance divided by the downward shortwave irradiance at each shortwave wavelength.	- Spectro-radiometer connected to cosine receptor or integrating sphere

**Table A4:** List of measurable properties associated with precipitating and suspended snow: name of the properties (first column), definition (second column), and existing instruments.

Parameter	Definition	Instruments
**Occurrence of drifting/blowing snow**	Drifting snow is defined as an ensemble of snow particles raised by the wind to small heights above the ground [67]. When the snow particles rise to a height of 1.8 m and above, the snow transport by the wind is called blowing snow [67]. The occurrence of drifting/blowing snow is recorded as presence or absence of it.	- Visual observation
**Height of new snow, or depth of snowfall (HN)**	Height of new snow is the vertical depth in centimeters of freshly fallen snow that accumulated on a snow board during a standard observing period of 24 h. Additional observation intervals can be used, but should be specified. For example, the notation HN(8h) or HN(2d) denotes an observation interval of 8 h or 2 days, respectively. The corresponding slope-perpendicular measurement is denoted by DN [14]. Special precautions should be taken so as not to measure old snow. This can be done by sweeping a suitable patch clear beforehand or covering the top of the snow surface with a piece of suitable material (such as wood, with a slightly rough surface, painted white) and measuring the depth down to this [34].	- Stakes - Rulers - Snow probe - *MagnaProbe* - Ultrasonic and laser depth sensors - Time-lapse photography - Terrestrial and air-borne laser scanner - Photogrammetry
**Hydrometeor fall velocity and size distribution**	Measurement of particle size distribution (PSD) and vertical fall velocity of the hydrometeors as a function of the diameter. The definition of the measured diameter varies among different instruments. For this reason, often the definition of diameter, detectable size range, and observation directions (e.g., 1D, 2D, and view angle) are given. PSD is typically integrated over a time interval (e.g., a one-minute-time interval).	- Optical disdrometer
**Number flux of drifting/blowing snow**	It is defined as the number of snow particles flowing through a unit area per second.	- Snow particle counter - Mechanical trap + wind speed measurements - Camera system with light source - *FlowCapt* - Optical disdrometer
**Particle size of drifting/blowing snow**	It is defined in the same way as snow grain size (see Snow microphysical properties).	- Sticking slides + photography + image processing - Camera system with light source + image processing - Disdrometer
**Precipitation Intensity**	The intensity of precipitation is defined as the mass flux over a given interval assuming a constant intensity. It is typically expressed in terms of depth per unit time, for example, millimeters per hour.	- Snow gauge - Present weather detector based on extinction

**Table A5:** List of measurable properties associated with snow composition: name of the properties (first column), definition (second column), and existing instruments.

Parameter	Definition	Instruments
**Snow impurity**	The type of impurity should be fully described and its amount given as mass fraction (%, ppm). Common impurities are dust, sand, soot, acids, and organic and soluble materials.	- Ion chromatography - Organic and elemental carbon analyzer (OC/EC) - *Single Particle Soot Photometer* (SP2) - Filter + spectrophotometer
**Snow isotopes**	The most relevant stable isotopes for water are ^18^O for oxygen (corresponding to the most abundant isotope ^16^O), and ^2^H (or Deuterium, D) for hydrogen (corresponding to the most abundant isotope ^1^H). The respective heavy water molecules are then $H_{2}^{18}O$ and HDO. Generally, stable isotopes are quantified as relative ratios of rare toward abundant isotope abundance, with respect to a mean standard (the Vienna Standard Mean Ocean Water [V-SMOW]), denoted with the symbol δ and expressed in per mille. Thus, snow isotopic composition is reported as δD, δ^18^O, and deuterium excess *d*.	- Mass spectrometer - Laser absorption methods (cavity ring down-spectroscopy-analyzer, integrated cavity output spectroscopy-analyzer)

## Appendix B. List of the Instruments Applied to Measure the Snow Properties

The instruments applied to measure snow properties are listed in Table A6, in alphabetic order. The first column includes the name and the classes of the instruments, where the classes are defined following Kinar and Pomeroy [15] and Section 3.3: portable/stationary, invasive/non-invasive, passive/active, manual-without-electronic/manual-with-electronic/automatic. Here, for invasive, we intend that the instrument modifies some snow properties at the time of the measurements, while we have classified as non-invasive those instruments that do not enter in direct contact with the target snow, or that are placed on the ground before snow accumulates on them and are partly or totally buried into the snowpack while recording the measurements. The devices that are unique (i.e., developed by a single group or company and usually known by their name), are written in italics, and the name of their manufacturer is provided when available. In the second column, a description of the measurement principle is given, and in the third column the measured or derived snow properties are listed (properties indirectly derived from other measured quantities are marked in italic).

**Table A6:** List of instruments, in alphabetic order.

Instrument (Classification)	Description	Measured or derived snow properties (derived properties are marked in italic)
**Acoustic sensor for snow bulk properties**	The sensor is composed of one or more microphones and a loudspeaker (e.g., [68,69]). It is placed above the snow surface and sends a sound wave into the snow. The bulk and thermal properties of the snow influence the speed and attenuation of the sound waves propagating through the snowpack and reflected to the microphone(s). Through an acoustic model, the reflection of the sound wave is related to the bulk and thermal properties of the snow.	- *SWE* - *Snow density* - *LWC* - *temperature*
(portable or stationary, non-invasive, active, manual with electronics or automatic)		
**Acoustic snow depth sensor**	See **Ultrasonic snow depth sensor**	
***ASSSAP***	The device is a 1 m long cylinder with a 10 cm diameter, inserted into a pre-drilled hole in the snowpack, to measure the SSA profiles. The optical head is constituted of an infrared laser diode at 1310 nm wavelength, which illuminates the snow horizontally (i.e., perpendicularly to the snow surface), following the design of a previous instrument called POSSSUM [12]. The radiation reflected back by the snow is recorded by six InGaAs photodiodes located on a crown at different viewing zenith angles. The distance between the snow surface and the photodiodes is measured with a 810 nm laser diode and a silicon photodiode receiver. The signals measured by the photodiodes are converted to bidirectional reflectance through a calibration fit, obtained using several reflectance standards. SSA is finally obtained from the radiation measurements using a model-based relationship.	- *SSA*
(portable, invasive, active manual with electronics)		
**Calorimetry**	Freezing, melting, and chemical calorimeters are used for measuring the LWC of snow. A calorimeter is an insulated container where the snow sample of a known mass is placed and temperature is recorded. A liquid is added to the container, and the change in heat content due to a change in phase of the mixture is recorded. The measured heat is related to the LWC of the snow sample, using a heat balance equation [14]. The added liquid can be a freezing agent, hot water, or a chemical agent.	- LWC
(portable, invasive, manual with electronics)		
**Camera**	See **Photography**	
**Camera system with light source**	The measurement system consists of a light source and a camera, horizontally aligned one in front of the other. The camera records shadow images of the drifting snow particles that pass through the illuminated volume (e.g., [70,71]). The measurement principle is the same as for a disdrometer, but the camera system is designed to measure horizontal particle flows (such as drifting snow) instead of vertical flows (such as solid precipitation).	- Particle size of drifting snow - Number flux of drifting snow
(stationary, non-invasive, active, automatic)		
**Cosmic ray sensor**	See **Neutron probe**	
***Denoth Meter***	It uses capacitance to measure the dielectric permittivity of the snow, which is related to its porosity and/or volumetric water content. It is a portable device consisting of a flat plate antenna that is inserted into the snowpack [72]. The plate can also be buried in the snowpack and used to measure diurnal cycles in snow wetness. Snow density and LWC are derived from the measured dielectric permittivity through empirical relationships.	- Snow dielectric permittivity - *LWC* - *Snow density*
(portable or stationary, Invasive, active, manual with electronics or automatic)		
**Dilution method**	The method consists in the dilution of snow samples extracted from the snowpack with an acid, which changes the concentration of ions, depending on the LWC. The conductivity of the resulting solution is measured with an electrolytic conductivity meter and is related to the snow LWC [73,74].	- Snow electrical conductivity - *LWC*
(portable, invasive, manual with electronic)		
**Disdromete**	Optical disdrometers are employed for the detection of falling snow particles. Typically, a light source (laser beam or halogen lamp) and a light detector (photo diode or camera) are placed horizontally opposite to each other. The instrument records the light attenuations (shadows or images) of the particles as they fall through the observation volume (e.g., [75,76,77,78,79]). The diameter of the particles is calculated (e.g., from the reduction of electric voltage on a photo diode or with optical imaging software depending on the instrument). The fall velocity is retrieved from multiple, consecutive records. The number of the particles is obtained from the amount of adequately measured particles over the observed time period.	- Hydrometeor fall velocity - Particle size distribution - Particle size of drifting/blowing snow - Number flux of drifting snow
(stationary, active, automatic)		
***FlowCapt*** **anemo-driftometer (ISAW, Tannay, Switzerland)**	The sensor determines both the wind velocity and snow particle flux. The detection principle is based on mechanical-acoustic coupling. The sensor is composed of closed pipes containing electro-acoustic transducers and a powering, filtering, and amplifying unit. When the sensor is placed into a particle flux, the particles shock the sensor pipes, inducing acoustic pressure. The pressure is picked-up by the transducers. The electrical outputs are filtered and time-averaged in given frequency ranges, to provide a signal proportional to particle flux [80].	- Wind velocity - Snow particle flux
(stationary or portable, non-invasive, automatic)		
**Foot penetration**	It is measured with a step into undisturbed snow, by putting the full body weight on one foot. The depth of the generated footprint, measured to the nearest centimeter from 0 to 5 cm, and, thereafter, to the nearest increment of 5 cm [81], indicates the surface penetrability. Foot penetration is sensitive to the weight of the observer.	- Surface penetrability
(portable, manual, invasive)		
**Gamma ray sensor**	A gamma sensor is constituted by a passive detector installed above the snow surface that measures the natural background radiation emitted by the soil and attenuated by the snowpack above it [82]. The attenuation of the gamma radiation is calculated with respect to the measurements done during the snow-free season, and an empirical equation is used to relate it to SWE [15].	- *SWE*
(stationary, non-invasive, passive, automatic)		
**Global Positioning System (GPS) Reflectometry**	Ground-based GPS receivers installed above the snowpack detect interferences between the direct signal from the GPS constellation, the reflected signal by the snow surface and possibly by other paths through the snowpack. As the multiple reflections of electromagnetic waves from the snow affect the signal-to-noise ratio (SNR) at the GPS receiver antenna, changes in SNR are related to surface height as well as the bulk properties of the snowpack. Using empirical or physically-based models of the propagation of electromagnetic radiation in the snow, snow macrophysical properties can be estimated from the SNR (e.g., [38,39,40]).	- *HS* - *SWE* - *Bulk snow density* - *Liquid water content*
(portable and/or stationary, non-invasive, active, automatic)		
**Gonio-spectro-radiometer**	Gonio-spectro-radiometers are spectro-radiometers (see Spectro radiometers) measuring via fiber optics and narrow-field-of-view optical lens (e.g., 3°). The optical lens is installed on a moving arm, which rotates in both zenithal and azimuthal directions, to sample the snow reflectance from the whole hemisphere. The movement is such that the sensor is viewing the same target point on the snow at any time and from every position. The reflectance is calculated from the ratio between the signal received from the snow and that received from a white reference (see e.g., [83,84,85]).	- *Snow BRDF* - *Snow spectral albedo*
(portable, non-invasive, passive, manual with electronics or automatic)		
**Ground Penetrating Radar (GPR)**	GPR sends an electromagnetic wave towards the snow and receives the reflected signal. The snowpack bulk properties are calculated from the reflected signal using empirical relationships or physically-based models of the propagation of electromagnetic radiation in the snow. Higher frequency radars have a better vertical resolution but they are subject to greater attenuation resulting in a lesser depth penetration than lower frequency radars. The latter, on the other hand, are more appropriate for the highly scattering wet snowpack (e.g., [86]). Upward looking GPR at the ground surface combined with GPS receivers can retrieve LWC, HS, and SWE continuously and independently [87]. Non-imaging, accurately calibrated radars are called scatterometers.	- *HS* - *SWE* - *Bulk snow density* - *LWC* - *Snow accumulation* - *Microwave-equivalent snow grain size*
(portable and/or stationary, non-invasive, active, automatic)		
**Hand test**	The Hand test is made by the observer to measure the hardness of snow layers in a snow pit. The observer pushes a fist, four fingers, a finger, a pencil, or a knife (in order of increasing hardness) into the snowpack. The hardness is recorded as the element that can be inserted into the snow [14]. The accuracy varies between observers [88].	- Hardness Index
(portable, invasive, manual)		
**Heat flux plate**	Heat flux plates (HFP) are small (often <50 mm per side), thin (<5 mm thick), rigid sensors placed horizontally in the snow at the desired depth(s). Most employ a thermopile to measure the temperature difference between the top and bottom of the plate [89]. The thermal conductivity is derived from the temperature gradient and the measured heat conduction flux between the snow below and above the plate. Modern HFPs are always guarded [90].	- Heat flux - *Snow thermal conductivity*
(portable and/or stationary, invasive, automatic)		
***HOBO Pedant temperature data logger*** **(Onset Computer Corporation, Buorne, MA, USA)**	It is a miniature data logger that can record temperature. It has a waterproof casing and use solar radiation shield for accurate temperature measurements in sunlight. It has a changeable battery. As it does not transmit the data externally, it is usually buried into the snow at known depth over one or more seasons and later recovered to download the data.	- Snow temperature
**iButton**	An iButton is a computer chip that is enclosed in a 16 mm thick durable weather resistant stainless steel can. It integrates a globally unique address, a digital thermometer, a thermal history log, an alarm event log, and additional memory to store user data. As it does not transmit the data externally, it is usually buried into the snow at a known depth over one or more seasons, and later recovered to download the data. The device can be used until the internal battery is consumed. The battery is not rechargeable or replaceable.	- Snow temperature
(portable or stationary non-invasive automatic)		
***IceCube,*** **originally called *DUFISSS* (A2 Photonic Sensors, France)**	About 4 or 8 cm^3^ of snow sampled from a snow pit wall is collected into a black sample holder, which is place inside an integrating sphere [11]. An infra-red laser diode illuminates the snow sample, and the reflected light is measured by an InGaAs photodiode placed at the output port of the sphere. Reference targets are used to determine the empirical fit that relates the photodiode signal to reflectance. The SSA is then derived from the reflectance through a fitting curve calculated using SSA direct observations obtained with methane absorption method [11].	- *SSA*
(portable, invasive, active, manual with electronics)		
**Infrared sensor (or camera) above the surface**	Infrared temperature sensors and cameras measure the longwave radiation emitted by the snow surface. The surface temperature is inferred by applying the Stefan–Boltzmann law, which relates the temperature of a black body to its emitted longwave radiation, and by assuming a certain snow emissivity.	- *Snow surface temperature* - *Snow presence*
(portable or stationary, non-invasive, passive, and automatic)		
**Infrared sensor, Probe**	A handheld infrared sensor probe can be applied to quickly measure the vertical profile of snow temperature in a snowpit, immediately after the cut of the wall.	- Snow temperature
(portable or stationary, non-invasive, passive, manual with electronics or automatic)		
***InfraSnow***	The *InfraSnow* instrument measures optical equivalent grain size, which can be converted to specific surface area [29]. The instrument uses a light-emitting diode with a wavelength of 950 nm and a photodetector. The very portable instrument is used to quantify the snow properties on ski tracks [91]. The longer wavelength, compared with the IceCube and ASSSAP, leads to a reduced sensitivity to surface sample disturbance. The opening of the instrument is directly put on the snow surface, the only requirement is that the surface is flat, but no other snow preparation is necessary. This simple application comes with the drawback that the intensity of reflection is somewhat density dependent, so an estimate or joint measurement of the snow density is necessary.	- *Optical equivalent grain size* - *SSA*
(portable, non-invasive, active manual with electronics)		
**Ion chromatography**	Ion chromatography separates ions and polar molecules based on their affinity to the ion exchanger. The snow sample is dissolved in a fluid called the mobile phase, which carries it through a structure holding another material called the stationary phase. The stationary phase is positive or negative charged, and ions of the various snow constituents travel at different speeds, resulting in the separation of the constituents [92].	- Snow impurity content
(invasive, manual with electronics)		
**Laser absorption methods**	Snow is transformed into the gas phase, placed into a cavity, and illuminated with a laser beam. A photodetector measures the gas absorption spectrum, which reveals the concentration of the chemical elements composing the snow. In cavity-ring-down spectroscopy, the laser beam is entered into the cavity and starts bouncing between mirrors. Once the light source is switched off, the time of decay, or ‘ring down’, of the intensity of the beam depends on the snow isotopic composition.	- *Snow isotopes*
(invasive, active manual with electronics)		
**Laser depth sensors**	Laser sensors are based on the measurement of time between the sent and received electromagnetic laser pulse, the latter being reflected from the ground/snow surface. The difference between the measurements taken in snow-free and snow-covered scenes allows the calculation of depths [93].	- *HS* - *Snow presence*
(stationary, non-invasive, active, automatic)		
**Laser scanner (ground based and airborne)**	The laser scanner (or lidar) measures the time between the sent and received laser pulse, the latter being reflected from the ground/snow surface. The scanner/laser beam moves horizontally and/or vertically to scan a line. Differencing co-registered lidar maps from two dates (snow-off from snow-on) allows the calculation of HS with sub-decimeter vertical uncertainty and high horizontal spatial resolutions over spatial extents compatible with basin-scale hydrologic needs and for slope-scale investigations (e.g., [94,95]). Time-lapse laser scanning also exists on a small scale [96]. Snow surface roughness can be derived from distance differences along the measurement line(s).	- *HS* - *Snow surface roughness*
(portable and/or stationary, non-invasive, active, manual with electronics)		
**Lidar**	See **Laser scanner**	
**Loaded column test**	The loaded column test allows an observer to estimate how much additional mass a weak layer might support before it will fracture. A snow column is isolated with a saw, and excavated blocks of snow with same area are stacked on the column until it fractures. The level of the fracture is recorded and the mass of each block can be measured. The shear resistance is derived from the measurement of the total load causing the failure of the column [81].	- Snowpack stability index - Snow strength/shear resistance
(portable, invasive, manual)		
**Lysimeter**	The lysimeter measures the amount of water melting from a snowpack (runoff). It consists of a collection pan which leads the water to a measurement gauge through a pipe or orifice [15].	- Runoff
(stationary, non-invasive, automatic)		
***MagnaProbe*** **(Pasco, CA, USA)**	Self-recording snow depth probe developed by [97], which uses a sliding electromechanical basket that reaches the snow surface when the tip of the pole contacts the ground surface. The distance between the plate at the snow surface and the tip of the pole at ground is measured electronically by pushing a switch. A GPS system provides the geographical location of each point. Time, location, and HS can be recorded electronically using standard loggers [15].	- HS
(portable, non-invasive, manual with electronics)		
**Magnifying glass**	The snow crystals are collected above a gridded black plate and carefully disaggregated with a spatula or a sharp needle. The magnifying class is then placed above the plate, and through it the observer obtains a visual estimation of the snow grain size and shape [14].	- Snow grain size - Snow grain shape
(portable, invasive, manual)		
**Mass spectrometer**	The snow samples are first melted and diluted with acid and then nebulized in the mass spectrometer. There, the gas molecules are fragmented into ions through electron ionization. The ions are separated in the mass spectrometer according to their mass-to-charge ratio, and are detected in proportion to their abundance. A mass spectrum of the molecule is thus produced and the Pb isotope concentration determined (e.g., [98]).	- *Pb isotopic composition in snow*
(Portable, invasive, active, manual with electronics)		
**Mechanical trap + wind speed measurements**	The mechanical trap is used to measure the blowing snow mass flux [15]. It consists of a collection container or a fabric bag with an orifice. The container is oriented toward the wind to collect the snow particles. The measurement of the mass of the collected snow particles is done by manually emptying the container and weighing its content or by applying an electronic weighing scale directly connected to the container, and it enables the calculation of the mass flux of blowing snow. With concomitant measurements of wind speed (with an anemometer), the flux density (number flux) of blowing snow particles crossing a unit area in 1 s can be calculated [15].	- Mass flux of drifting/blowing snow - Number flux of drifting/blowing snow
(Portable or stationary, manual or automatic)		
**Methane absorption method**	The method consists in placing a snow sample in a container with CH_4_ at the temperature of 77 K, and measuring the number of CH4 molecules absorbed by the snow (i.e., that can be accommodated on the snow surface). Knowing the cross sectional surface area of a CH_4_ molecule, the area of the snow sample that is accessible to gases can be determined by BET analysis. SSA is obtained dividing the area of the snow sample by its weight [99,100,101].	- *SSA*
(lab-based, manual with electronics)		
**Micro-computed tomography (micro-CT)**	The micro-CT instrument takes X-ray scans (images) of a snow sample, each scan reproducing a slice of the sample. After the three-dimensional image of the microstructure of the snow sample is reconstructed from the scanned slices, an image processing software is applied to derive the stereological properties characterizing the snow sample. The measurements can be done using natural snow sample [102] or cast snow samples [103]. Though numerical simulations, several snow properties can be derived from the observed micro-CT images [104].	- Snow correlation length - Snow grain shape - Snow porosity - SSA - Snow tortuosity - Air permeability of snow - *Snow stickiness* - *Snow thermal conductivity*
(lab-based, manual with electronics)		
**Microwave radiometer**	A ground-looking microwave radiometer measures the microwave radiation (at frequencies of 1–1000 GHz) emitted from the ground and the snow, which is called the brightness temperature. Microwave radiometers are usually equipped with multiple receiving channels, which detect a signal originating from snow/ground layers of different thickness (e.g., [22]). From the measurements of snow brightness temperature, is it possible to retrieve the snow cover extent, HS, SWE, melt events, snow temperature, and microwave-equivalent snow grain size (e.g., [20,105,106]).	- Snow brightness temperature - *Snow cover extent* - *HS* - *SWE* - *Snow temperature* - *Microwave-equivalent snow grain size*
(portable/stationary, non-invasive, passive, automatic)		
**Multichannel Filter Radiometer (MCFR)**	MCFRs measure UV/solar radiation in several discrete wavelength bands. The fore-optic generally consists of a diffuser, the angular response of which should ideally be proportional to the cosine of the zenith angle. The wavelength selection is typically achieved by narrow to moderate band interference filters, and the signal is detected with a photodiode or a phototube (without dynodes for multiplication). Compared to spectroradiometers and broadband instruments, interpretation of data of these instruments is more complex and the separation of instrument characteristics and data products is not straightforward [107]. The snow UV/multichannel albedo is obtained using a pair of upward and downward looking MCFRs (e.g., [108]).	- Snow multichannel albedo
(portable, non-invasive, passive, and automatic)		
**Near-Infrared (NIR) photography**	In NIR photography, a digital camera filtered to detect the 850–1000 nm wavelength range is applied to distinguish snow layers of different microstructure. The method consists of taking a photo of a smoothened snow pit wall, where also a ruler and/or plates of known reflectance are placed for reference. The snow stratigraphy is directly obtained from the visual assessment of the photo, and the SSA of each layer is derived from the measured reflectance through an empirical fit [28,109].	- Layer thickness (stratigraphy), - *SSA* (or optically equivalent grain size)
(portable, invasive, passive, manual with electronics)		
**Needle probe**	See **Transient needle probe**	
**Neutron probe**	A neutron probe (or cosmic-ray probe) measures the intensity of neutrons received from the surface. Neutrons generated by cosmic rays near the earth surface are slowed through collisions with the hydrogen nuclei of water molecules [110]. Thus, the moderation of neutron intensity measured by the neutron probe above the snow is negatively correlated with the SWE [111]. The footprint of the probe is few hundred meters (depending on air pressure). Bulk snow density can be derived with a co-located HS sensor [112].	- *SWE* - *Snow density*
(portable or stationary, non-invasive, passive, automatic)		
**Organic carbon (OC) and elemental carbon (EC) analyser**	Many different thermal, optical, and thermal/optical carbon analysis methods for organic carbon (OC), elemental carbon (EC), or black carbon (BC) have been applied throughout the world [113]. The snow impurity content of melted snow samples is collected in filters. OC and EC are measured by thermal evolution methods that quantify the amount of carbon that leaves the filter at different temperatures [114]. These methods use different combinations of temperature and analysis atmospheres to evaporate, pyrolyze, and combust the carbon-containing compounds on a filter sample, and then detect the evolved carbon gases. The separation of OC from EC is ambiguous because some of the EC combusts in the presence of oxygen, and some of the OC chars (turns to EC) in an oxygen-deficient atmosphere. Light reflected from or transmitted through the filter during the analysis is used to monitor and correct for this charring [113].	- *Content of elemental carbon (soot, black carbon) and organic carbon in snow*
(lab-based, invasive, manual with electronics)		
**Permeometer**	Permeometer is used to measure the permeability of snow [115]. As reported in [15], the snow sample is placed inside a container, and air is pumped through the sample. The speed of the air and the air pressure inside and outside of the container is recorded for different air flow rates. The air saturated permeability is then determined through a regression equation [115].	- *Air permeability of snow*
(portable, invasive, automatic)		
**Photogrammetry**	See **Photography**	
**Photography (or photogrammetry)**	Digital camera and video cameras are employed to do snow photogrammetry (i.e., to measure snow properties). A large variety of snow properties can be measured applying different setups and image processing algorithms. Time lapse-photography with cameras or webcams at fixed position is applied to retrieve snow covered area (e.g., [41,116]), snow presence, HS, and height of new snow (e.g., [117,118,119]). Cameras mounted on manned or unmanned aerial vehicles allow the mapping of HS [120,121,122] and snow BRDF [123], the latter requiring calibrated white targets at the surface as a reference. Snow surface roughness is obtained from the processing of photos with reference scales, such as a graduated blackboard vertically inserted into the snow surface [124] and graduated survey strings aligned over the glacier surface [125]. Macro-photography is applied to measure snow grain shape and size metrics (as the optical equivalent snow grain size) of snow particles extracted from the snow pit wall (e.g., [30,31]) and of drifting snow particles attached to sticking slides (e.g., [126]).	- *Snow covered area* - *Snow presence* - *HS* - *Height of new snow* - *Snow surface roughness,* - *Snow grain shape* - *Snow grain size according to different metrics* - *BRDF* - *Particle size of drifting/blowing snow*
(portable or stationary, invasive or non-invasive, manual with electronic or automatic)		
**Present weather detector (PWD)**	The PWD is a multi-variables sensor for automatic weather observing systems. It combines an optical sensor, a capacitive rain sensor, and a Pt100 thermistor. The working principle of the optical sensor is similar to the optical disdrometer (see Disdrometer). However, the PWD is designed to detect the bulk characteristics of the precipitation, without resolving the single precipitation particles. PWD are usually operational instrument, providing products that are needed for operational applications, such as aviation and road weather, while optical disdrometers are applied more in research. Type of precipitation (fog, rain fall, snowfall, etc.), visibility, and precipitation intensity are calculated from the attenuation (extinction) of the light emitted by a laser and detected by photodiodes when the precipitating particles fall through the observation volume [127]. The optical sensor can use the principle of forward scattering or extinction. The capacitive sensor measures the presence and amount of water on its surface. The signal is dependent on the thickness of the water on the sensor, which is a measure of the liquid water content of the precipitation. The Pt100 thermistor is used to monitor the temperature, as an adjustable parameter for classification of precipitation types.	- *Type of precipitation* - *Precipitation intensity* - *Visibility*
(Stationary, non-invasive, active, automatic)		
**Propagation saw test (PST)**	The PST tests the propensity of a pre-identified deeply buried weak-layer and slab combination to propagate a crack in the snowpack, independently of any loading for fracture initiation [128,129]. The tester uses an isolated column and initiates a fracture by dragging a snow saw along the weak layer in the uphill direction. The tester should describe the way the fracture propagates ahead of the saw according to a coded description.	- Fracture propagation - *Snow strength/shear resistance (qualitative)*
(portable, invasive, manual)		
**Push–pull gauge**	The push–pull gauge measures horizontal compressive force and consists of a load cell connected to a bolt; when the bolt is pushed, the force is measured and is shown directly on a display (e.g., [130,131]). Different attachments can be connected to the push–pull gauge. The force recorded when the attachment is pushed against the snow pit wall can be converted to hardness index using the Table 1.4 of Fierz et al. [14].	- *Hardness*
(portable, invasive, manual with electronics)		
**Pyrgeometer**	A pyrgeometer measures the broadband longwave flux in the wavelength range from 4.5 to 100 μm received from its hemispherical field of view. A couple of upward- and downward-looking pyrgeometers installed about 2 m above the surface is needed to measure the snow surface net longwave flux (sum of flux emitted by the snow surface and received from the overlying atmosphere). The snow surface temperature is inferred from the net longwave flux by applying the Stefan-Boltzmann law, which relates the temperature of a black body to its emitted longwave radiation, and by assuming a certain snow emissivity.	- Snow surface upward/net longwave flux - *Snow surface temperature*
(stationary, non-invasive, passive, automatic)		
**Pyranometer**	A pyranometer measures the broadband shortwave flux in the solar wavelength range (ca. 0.3 μm to 3 μm) from its hemispherical field of view. In thermopile pyranometers, the solar radiation absorbed by a black surface heats the thermopile junction located beneath it and generates a small voltage, which is proportional to the absorbed shortwave irradiance. A couple of upward- and downward-looking pyranometers, installed about 2 m above the snow surface are needed to calculate the snow broadband albedo from the ratio between the upward and downward shortwave fluxes.	- Incoming/reflected broadband irradiance - Snow broadband albedo
(stationary, non-invasive, passive, automatic)		
**Radar**	For radars applied in near-field (in-situ) observations (see Ground-penetrating Radar).	
***Rammsonde***	The *Swiss Rammsonde* consists of a cave rod, with a 60° cone tip angle, to which is attached a drop hammer. The hammer is dropped from a known height through a thin guide inside the rod, causing the penetration of the cone into the snowpack [132]. Knowledge of the hammer height before the drop, the mass of the hammer, and the penetration depth, enables the computation of the ram resistance.	- Hardness - Surface penetrability
(portable, invasive, manual with/without electronics)		
**Reflectometry**	See **GPS Reflectometry**	
**Rutschblock test**	The Rutchblock (or glide-block) test was developed in Switzerland. A snow column is isolated by hand using a saw [133], and its collapse after a series of loading steps by the skier is recorded with the appropriate Rutschblock score [81]. The test is mainly used for snow stability in slopes.	- Snowpack stability index - *Snow strength/shear resistance (qualitative)*
(portable, invasive, manual)		
**Scale**	See **Snow scale**	
**Scatterometer**	See **Ground-penetrating Radar**	
**Shear frame test**	The shear frame test is used to measure the shear strength of snow layers and of the interfaces between them. The shear frame consists of a metal frame with parallel cross bars [134]. A force gauge is attached to side of the frame, and the observer pulls on the frame so that fracture in the snowpack occurs within 1 s [81]. The shear strength is calculated from the average measured shear force, and it depends on the frames size.	- Snow strength/shear resistance
(portable, invasive, manual)		
**Shovel Shear Test**	Shovel shear test is made to detect unstable layers in deep snow over slopes. A snow column is isolated from the snowpack through vertical cuts on the downslope and lateral sides. A vertical cut of the depth of the shovel plate is made on the back (upslope) side of the column. Shovel is inserted into the cut and gently pulled in the downslope direction until the column breaks. The depth of the shear plane where the break occurred is recorded, and the procedure is repeated until the ground is reached [81].	- Snowpack stability index - *Snow strength/shear resistance (qualitative)*
(portable, invasive, manual)		
***Single Particle Soot Photometer (SP2)*** **, (Droplet measurement technologies, Inc., CO, USA)**	SP2 is used to analyse the black carbon (BC) concentration in snow and ice. Unless filter-based methods (see organic carbon [OC] and elemental carbon [EC] analyser), SP2 does not need a filtration step, and operates with very little sample volume (e.g., [135]). However, the SP2 requires an aerosolization step, because it analyzes only air-borne samples. A laser heats the individual BC particles, and the instrument detects the thermal radiation emitted by them when they become incandescent. This signal is proportional to the BC mass [136]. Thus, SP2 provide continuous, automatic measurements of mass concentration and size distribution of BC on a particle-by-particle basis [135].	- Snow impurity (BC) concentration and size distribution
(portable, invasive, automatic)		
**Ski penetration**	It is measure with a step into undisturbed snow, by putting full body weight on one ski. This generates a ski track, the depth of which is measured from the centreline to the nearest centimetre from 0 to 5 cm, and, thereafter, to the nearest increment of 5 cm [81], which indicates the surface penetrability. Ski penetration is sensitive to the weight of the observer and the surface area of the ski.	- Surface penetrability
(portable, manual, invasive)		
**Snow cutter**	The snow cutter is used to extract snow samples of a known volume from the snow pit walls to measure the vertical profile of snow density. The cutter may have different shapes (right-angled triangular prism, right rectangular prism, or cylinder), has sharpened edges, and is usually made of aluminium [137]. The cutter is horizontally or vertically inserted into the targeted snow layer and extracted with the help of a separate metal plate that sharply cuts one side of the sample. The mass of the snow sample is then weighed with a scale and the density is derived from its volume and weight. The SWE of the snowpack can be obtained from the snow density measurements if the whole vertical profile of the snowpack is sampled.	- Snow density - *SWE*
(portable, invasive, manual)		
***Snow Fork*** **(Insinööritoimisto Toikka, Finland)**	It is a microwave resonator used to measure the dielectric permittivity of the snow, which is related to its porosity and/or volumetric water content. It is a portable device consisting of two parallel stainless steel prongs with sharpened ends that act as transmission line and are usually inserted horizontally into the side of a snowpit [138]. Snow density and liquid water content are derived from the measured dielectric permittivity through empirical relationships.	- Snow dielectric permittivity - *LWC* - *Snow density*
(portable, invasive, active, manual with electronics)		
**Snow (or precipitation) gauge**	The snow gauge term is used to separate this gauge from the vast family of precipitation gauges, which are suitable to measure only rain. Snow gauge measures the amount of solid precipitation in a certain period of time, and typically the precipitation intensity is expressed as liquid water equivalent [139]. Falling snow is collected into a catchment container. The mass of the collected snow and time are recorded and converted to precipitation intensity. For the automatic snowfall observations, only the weighing-gauge type is suitable. Manual measurements are made by removing the container and melting the collected snow. For accurate snowfall measurements the gauge should have a wind shield, typically, in operational use, an Alter or a Tretyakov-types are utilized. The reference gauge for the solid precipitation measurements is surrounded by an octagonal double fence also known as the Double Fence Intercomparison Reference (DFIR, [140]).	- SWE of new snow - Solid precipitation intensity
(Stationary, not-invasive, manual or automatic)		
***SnowMicroPen***	The instrument is a high-resolution penetrometer, developed at the WSL Institute for Snow and Avalanche Research (Switzerland), which automatically measures the resistive force produced when a rode with in a conical sensitive tip penetrates perpendicularly into the snowpack at a constant speed [141]. The resistive force is related to the texture of snow, thus empirical relations are used to derive the vertical profile of snow density, hardness, SSA, correlation length, layer thickness, and HS [13].	- Resistance (force) to penetration - *Density* - *SSA* - *Correlation length* - *Layer thickness* - *HS*
(portable, invasive, manual with electronics)		
***Snowpack Analyzer (SPA)*** **(Sommer Meschtechnik, Koblach, Austria)**	It applies TDR (see in this table) to measure the dielectric constant of the snow [37], from which density and LWC are derived. It uses a flat coaxial cable antenna installed at an angle to the ground surface. The antenna is buried in the snowpack when snowfall accumulates. The assembly includes an acoustic sensor (see in this table) that measures HS and permits the calculation of SWE.	- Snow dielectricpermittivity - *SWE* - *LWC* - *Snow density* - *HS*
(stationary, non-invasive, active, automatic)		
**Snow particle counter (SPD)**	SPD is an optical device that measures the diameter and the number of drifting snow particles by detecting their shadows on a photodiode [142]. A laser diode is used as a light source, and a photodiode detects the interruption of the light beam by a blowing snow particle. The SPD is able to detect particles with diameter in the range of 40–500 μm.	- *Number flux of drifting/blowing snow* - *Mass flux of drifting/blowing snow* - *Snow particle size distribution*
(Stationary, non-invasive, automatic)		
**Snow pillow**	The snow pillow is a pressure transducer filled with antifreeze fluid installed at the ground surface under the snowpack [143]. Through the pressure changes of the fluid, the instrument measures the mass of the snow accumulated over the instrument, which gives the SWE once the pillow’s area is known.	- SWE
(stationary, non-invasive, automatic)		
**Snow probe**	The snow (or avalanche) probe is constituted of several graduated rigid sticks connected by strings that can quickly be joined together to form a single long rod. It is vertically inserted into the snow surface until it reaches the ground to measure the snow depth.	- HS - Height of new snow
(portable, invasive, and manual)		
**Snow ruler**	See **Snow stake and ruler **	
**Snow scale**	The snow scale is installed at the ground surface and automatically measures the weight and, therefore, the mass of the snow accumulated over it. The SWE is obtained by dividing the measured snow mass by the area of the instrument. When the height of the snowpack is independently measured, the snow density can be calculated from the ratio between mass and volume of the sampled snow column.	- SWE - Snow density
(stationary, non-invasive, automatic)		
**Snow stake and ruler**	The snow stakes are graduated rigid sticks, which can be vertically inserted into the snow surface until they reach the ground. They are suitable for mapping the snow height along transects or snow courses. Portable snow rulers are usually carpenter’s folding rulers temporally installed against the snow pit wall for HS and layer thickness measurements [15]. The stationary version of the snow stake/ruler is a graduated rigid stick installed on the ground before the start of the snow season. The graduated scale marked on it can be read visually or through a camera. The scale reading provides an estimation of the height of the snowpack. When a board is placed over the old snow, it can measure the height of snowfall accumulated above the board.	- HS - Height of new snow - Snow layer thickness
(portable or stationary, invasive or non-invasive, manual)		
**Snow tube**	Snow tube (or snow sampling tube) is used to extract samples of snow to measure the snow water equivalent and density [144]. The material, length, and design of the snow tubes varies, but generally the instrument is a cylinder with a serrated cutting edge, pushed through the snowpack until its edge reaches the ground. The tube with the extracted snow sample is weighed with a scale. SWE is derived from the weight and volume of the snow sample. If the snowpack is deeper than the height of the tube, vertically aligned multiple samples are taken. To derive snow density, a simultaneous measurement of HS is needed (e.g., [145]).	- SWE - Snow density
(portable, invasive, manual)		
**Spectrophotometer**	Spectrophotometers are applied to measure the amount of light that is absorbed by the impurity content of the snow. Snow samples are melted and filtered, and the filters are inserted into the spectrophotometer. A light source shines on the filters and the absorption spectrum is measured. The spectrum is then analyzed to infer the concentration of black carbon (BC), the fraction of absorption due to non-BC light-absorbing constituents and the absorption Ångstrom exponent of all particles [146].	- *Snow impurity concentration and characterization*
(portable, invasive, active, manual with electronics)		
**Spectro-radiometer**	Spectro-radiometers include one or multiple photodetectors that are sensitive to one portion of or to the whole solar spectrum. Depending on the connected light receptor (narrow-angle fore optic, diffuser for hemispherical light reception, integrating sphere for hemispherical light reception), they measure spectral radiance or irradiance. When facing the snow surface, they measure the spectrum of the radiance/irradiance reflected from the snowpack. The snow spectral reflectance/albedo is calculated from the ratio of reflected radiance/irradiance to the incoming radiance/irradiance. All of the commercially available spectro-radiometers that can detect the full solar spectrum are portable (manual) instruments (e.g., [147]). Few spectrometers that are applied for stationary and automatic measurements cover only part of the solar spectrum (e.g., [148,149]). Near-surface snow SSA and optical-equivalent snow grain size can be derived from the spectral reflectance/albedo measurements using radiative transfer models. Connecting a contact probe to the manual spectro-radiometer via a fiber-optic cable, it is possible to measure the vertical profile of SSA, e-folding depth, and optical transmittance from the wall of a snow pit (e.g., [150]). The same quantities can also be measured using several contact probes buried in the snow at different depth and connected to an automatic spectro-radiometer via fiber optic cables (e.g., [149]).	- Incoming/reflected spectral radiance - Incoming/reflected spectral irradiance - Snow directional reflectance - Snow spectral albedo - *Snow broadband albedo* - *SSA* and optically-equivalent snow grain size - *Snow optical transmittance* - *Snow e-folding depth*
(portable or stationary, invasive or non-invasive, passive, manual with electronics or automatic)		
**Sticking slides + photography and image processing**	See **Photography**	
**Tacheometry****(portable**, non-invasive manual with electronics)	Tacheometry is the combination of (horizontal and zenith) angle and (slope) distance measurements from a standing point to observation/target points (retro-reflectors). It allows the determination of a point’s coordinate in three dimensions by transferring polar measurements into a Cartesian coordinate system. This technique can be used for monitoring the HS on slopes.	- HS - Snow covered area
**Thermistor probe, Handheld or connected to data logger**	A thermistor probe measures the temperature through the temperature dependence of its resistance. The thermistor is incorporated in the stainless steel head of the probe, which is inserted into the snowpack. The data logger automatically converts the recorded resistance to temperature through a polynomial equation. It is usually inserted across the snowpit wall at different snow depths to measure the temperature profile of snowpits (e.g., [151]), but it can also be placed in stationary installations, as a single element or as array of probes at different heights that are buried into the snow after snow accumulation (e.g., at the Arctic Research Centre in Sodankylä, Finland: http://litdb.fmi.fi/ioa0007_data.php).	- Snow temperature - *Snow presence*
(portable or stationary, invasive or non-invasive, manual with electronics or automatic)		
**Thermistor string, Fixed**	A Thermistor string is cable with several thermistors installed at regular/known distances, which is buried into the snow to measure the vertical profile of the snow temperature. The measured snow temperature profile together with an independent estimation of the heat flux into the snow can provide the snow thermal conductivity.	- Snow temperature - *Thermal conductivity*
(stationary, non-invasive automatic)		
**Time Domain Reflectometry (TDR)**	The TDR method measures the return time of an electromagnetic pulse transmitted through a finite length cable or probe inserted into the snow. The return time of the pulse is affected by both the length of the cable or probe (travel distance) and the permittivity of the snow around it (propagation velocity). When the length of the cable is known, the snow permittivity can be determined [152], from which snow density and LWC can be derived.	- Snow dielectric permittivity - *LWC* - *Snow density*
(Portable or stationary, non-invasive, active, manual with electronic or automatic)		
**Time-lapse photography**	See **Photography**	
**Tipping bucket gauge**	See **Snow gauge**	
**Transient needle probe**	The transient needle probe is a thin needle inserted into the snowpack, which is heated up through an electrical current. The change in temperature during heating or cooling of the probe is sensed by a thermocouple, and depends on the thermal conductivity of the snow (e.g., [153]). The probe can be manually inserted into the snowpit wall, or buried into the snowpack throughout the snow season [154].	- Thermal conductivity - *Snow density*
(Portable or stationary, invasive or non-invasive, manual with electronics, or automatic)		
**Translucent profile**	Translucent profile is made to detect stratigraphy of snow. Two snow pits are excavated opposite to each other so that a very thin, vertically flat section of snow is left between them [155,156]. Light penetrating through the snow section reveals the layer boundaries.	- Layer thickness (stratigraphy)
(invasive, Manual)		
**Ultrasonic snow depth sensor**	An ultrasonic sensor is fixed to a mast of known height above the snow surface. It produces an inaudible (>20 kHz) acoustic pulse that is reflected from the snow surface. Measuring the time between the sent and received pulses enables the retrieval of HS using kinematics [15]. The concomitant measurement of air temperature is needed to calculate the speed of the sound in the air.	- *HS* - *Snow presence*
(stationary, non-invasive, active, automatic)		
**Wind speed vertical profile**	The vertical profile of wind speed is obtained from weather masts or soundings. It is applied to derive the snow aerodynamic snow roughness, or roughness length, which is the height at which the mean wind becomes zero when extrapolating the logarithmic wind-speed profile downward through the atmospheric surface layer.	- *Snow roughness length* (aerodynamic roughness)
(portable or stationary; non-invasive; manual with electronics in case of sounding; or automatic		

## Appendix C. Questionnaire

The purpose of the questionnaire is to make a survey on the measured snow parameters and the applied instrumentation/techniques. The questionnaire only addresses general information, and should be filled in just once for ALL of the measured parameters. For each parameter, the applied instrument should be selected among the available choices or specified with text in the free space.

**Personal Information**

Name and Surname *

Position *

Institute *

Country *

E-mail address *

**Measured snow parameter(s)**

Do you measure snow macrophysical parameters? *

(Snow covered area (or snow extent), snow presence, snow depth, snow water equivalent, snow liquid water content (or wetness), snow density, snow temperature, snow layer thickness (stratigraphy), snow hardness, penetrability of snow surface, snow strength (shear resistance), snow roughness length (aerodynamic roughness), snow surface roughness, runoff)

- Yes
- No

**Measured snow parameter(s)**

Which macrophysical parameters do you measure using which instruments? Please select ALL the parameter - instrument pairs you use for measurement.

For the descriptions of the parameters, click here: http://costsnow.fmi.fi/index.php?page=A1.

- Snow covered area (Snow extent) - Optical remote sensing
- Snow covered area (Snow extent) - Camera or web-cam
- Snow presence - Visual
- Snow presence - Camera or web-cam
- Snow presence - Snow depth sensor
- Snow presence - Thermometer
- Snow presence - Infrared sensor
- Snow depth (HS) - Stakes
- Snow depth (HS) - Rulers
- Snow depth (HS) - Snow probe
- Snow depth (HS) - MagnaProbe
- Snow depth (HS) - Ultrasonic and laser depth sensors
- Snow depth (HS) - Time-lapse photography
- Snow depth (HS) - Terrestrial and air-borne Laser Scanner
- Snow depth (HS) - Unmanned Aerial Vehicle & Photogrammetry
- Snow depth (HS) - Ground Penetrating Radar
- Liquid water content (LWC) - Melting/freezing calorimetry
- Liquid water content (LWC) - Alcohol calorimetry
- Liquid water content (LWC) - Dilution method
- Liquid water content (LWC) - Time Domain Reflectometry (TDR)
- Liquid water content (LWC) - Snow Fork
- Liquid water content (LWC) - Denoth meter
- Snow Water Equivalent (SWE) - Snow sampling tube
- Snow Water Equivalent (SWE) - Snow cutters & scale
- Snow Water Equivalent (SWE) - Snow pillow
- Snow Water Equivalent (SWE) - Scale (i.e., Sommer scale)
- Snow Water Equivalent (SWE) - Snow gauge
- Snow Water Equivalent (SWE) - Passive snow water equivalent sensor (i.e., Campbell CS 725)
- Snow Water Equivalent (SWE) - Snowpack analyzer
- Snow Water Equivalent (SWE) - Gamma and cosmic rays sensors
- Snow Water Equivalent (SWE) - Ground penetrating radar
- Snow Water Equivalent (SWE) - Acoustic sensor
- Snow Density - Snow sampling tube
- Snow Density - Snow cutter & scale
- Snow Density - Scale (i.e., Sommer scale)
- Snow Density - Neutron probe
- Snow Density - Snow MicroPen
- Snow temperature (Ts) - Manual thermistor probe
- Snow temperature (Ts) - Fixed thermistor string
- Snow temperature (Ts) - Pyrgeometer (only surface)
- Snow temperature (Ts) - Infrared sensor (or camera) above the surface
- Snow temperature (Ts) - Probe with infrared sensors
- Snow temperature (Ts) - i-buttons
- Snow layer thickness (stratigraphy) - Visual observations & ruler
- Snow layer thickness (stratigraphy) - NIR photography
- Snow layer thickness (stratigraphy) - Translucent profile
- Snow hardness - Hand test
- Snow hardness - Push-pull gauges
- Snow hardness - SnowMicroPen
- Penetrability of snow surface - Ram penetrometer
- Penetrability of snow surface - Foot penetration
- Penetrability of snow surface - Ski penetration
- Snow strength/shear resistance - Shovel Shear test
- Snow strength/shear resistance - Loaded column test
- Snow strength/shear resistance - Trapezoidal Tensile test
- Snow strength/shear resistance - Flat jack Shear test
- Snow strength/shear resistance - Shear frame test
- Snow strength/shear resistance - Rutschblock test
- Snow strength/shear resistance - SnowMicroPen
- Snow roughness length (aerodynamic roughness) - Wind profile
- Snow surface roughness - Laser scanner
- Snow surface roughness - Photography
- Runoff - Lysimeter
- Other:

**Measured snow parameter(s)**

Do you measure snow microphysical parameters? *

(snow grain shape, snow grain size, snow optical equivalent grain size, snow SSA, correlation function, correlation length, snow porosity, snow permeability, snow tortuosity, snow stickiness, snow thermal conductivity, snow electrical conductivity)

- Yes
- No

**Measured snow parameter(s)**

Which microphysical parameters do you measure using which instruments? Please select ALL the parameter - instrument pairs you use for measurement. *

For the descriptions of the parameters, click here: http://costsnow.fmi.fi/index.php?page=A1.

- Grain shape - Visual
- Grain shape - Visual & magnifying glasses
- Grain shape - Macro-photography & image processing
- Grain shape - Casting & Micro-computed tomography & image processing
- Grain size - Visual & scale
- Grain size - Visual & scale & magnifying glasses
- Grain size - Macro-photography & image processing
- Grain size - NIR-photography
- Grain size - Casting & Micro-computed tomography & image processing
- Optical-equivalent grain size - Contact probe of Spectro-radiometer & snow-radiative transfer model
- Optical-equivalent grain size - Spectro-radiometer & snow-radiative transfer model
- Microwave equivalent grain size - Microwave radiometers & snow microwave emission model
- Specific Surface Area (SSA) - IceCube
- Specific Surface Area (SSA) - ASSSAP
- Specific Surface Area (SSA) - NIR-photography
- Specific Surface Area (SSA) - SnowMicroPen
- Specific Surface Area (SSA) - Casting & Micro-computed tomography & image processing
- Specific Surface Area (SSA) - CH4 absorption
- Snow correlation function - Casting & Micro-computed tomography & image processing
- Snow correlation length - Casting & Micro-computed tomography & image processing
- Snow correlation length - SnowMicroPen
- Snow tortuosity - Casting & Micro-computed tomography & image processing
- Snow porosity - Casting & Micro-computed tomography & image processing
- Air permeability of snow - Permeameter, or permeability apparatus (controlled air flow through a snow sample)
- Air permeability of snow - Casting & micro-computed tomography
- Snow stickiness - Casting & Micro-computed tomography& image processing
- Snow thermal conductivity - Heat flux plate
- Snow thermal conductivity - Transient needle probe
- Snow thermal conductivity - Thermistor string
- Snow thermal conductivity - Micro-computed tomography & image processing
- Other:

**Measured snow parameter(s)**

Do you measure snow electromagnetic properties? *

(snow bidirectional reflectance distribution function, polarization of snow optical reflectance, snow directional reflectance, snow spectral albedo, snow broadband albedo, snow e-folding depth, snow optical transmittance, snow brightness temperature, snow backscattering)

- Yes
- No

**Measured snow parameter(s)**

Which electromagnetic properties do you measure using which instruments? Please select ALL the parameter - instrument pairs you use for measurement. *

For the descriptions of the parameters, click here: http://costsnow.fmi.fi/index.php?page=A1.

- Snow Bidirectional Reflectance Distribution Function (BRDF) - Gonio-spectro-radiometer
- Snow Bidirectional Reflectance Distribution Function (BRDF) - Camera + white Lambertian-reflecting target
- Polarization of snow optical reflectance - Spectro-radiometer
- Snow Spectral Albedo - Spectro-radiometer connected to cosine receptor or integrating sphere
- Snow broadband albedo - Spectro-radiometer connected to cosine receptor or integrating sphere
- Snow broadband albedo - Pyranometers
- Snow e-folding depth - Fiber-optic probes
- Snow optical transmittance - Array of spectro-radiometers (two or more)
- Snow brightness temperature - Microwave radiometer
- Snow backscattering - Radar
- Snow electrical conductivity - Snow fork
- Other:

**Measured snow parameter(s)**

Do you measure snow solid precipitation? *

(height of new snow, precipitation intensity, drifting snow occurrence, number flux and particle size distribution of drifting snow)

- Yes
- No

**Measured snow parameter(s)**

Which solid precipitation properties do you measure using which instruments? Please select ALL the parameter - instrument pairs you use for measurement. *

For the descriptions of the parameters, click here: http://costsnow.fmi.fi/index.php?page=A1.

- Height of new snow, depth of snowfall - Stakes
- Height of new snow, depth of snowfall - Rulers
- Height of new snow, depth of snowfall - Snow probe
- Height of new snow, depth of snowfall - MagnaProbe
- Height of new snow, depth of snowfall - Ultrasonic and laser depth sensors
- Height of new snow, depth of snowfall - Time-lapse photography
- Height of new snow, depth of snowfall - Terrestrial and air-borne Laser Scanner
- Height of new snow, depth of snowfall - Photogrammetry
- Precipitation Intensity - Weighting-recording gauge
- Precipitation Intensity - Present weather detector based on extinction
- Hydrometeor fall velocity and size distribution - Optical disdrometer
- Drifting snow occurrence - Visual observation
- Number flux of drifting snow - Snow particle counter
- Number flux of drifting snow - Mechanical trap & wind speed measurements
- Number flux of drifting snow - Camera system with light source
- Number flux of drifting snow - FlowCapt acoustic sensor
- Number flux of drifting snow - Disdrometer
- Particle size of drifting snow - Sticking slides & photography & image processing
- Particle size of drifting snow - Camera system with light source & image processing
- Particle size of drifting snow - Disdrometer
- Other:

**Measured snow parameter(s)**

Do you measure snow composition? *

(snow impurity, snow isotopes)

- Yes
- No

**Measured snow parameter(s)**

Which snow component do you measure using which instruments? Please select ALL the parameter - instrument pairs you use for measurement.

For the descriptions of the parameters, click here: http://costsnow.fmi.fi/index.php?page=A1.

- Snow impurity - Ion chromatography
- Snow impurity - Organic and Elemental Carbon analyzer (OC/EC)
- Snow impurity - Single Particle Soot Photometer (SP2)
- Snow impurity - Filter & spectrophotometer
- Snow isotopes - Mass spectrometer
- Snow isotopes - Laser absorption methods (Cavity ring down-spectroscopy-analyzer, Integrated cavity output spectroscopy-analyzer)
- Other:

**Landscape**

Where do you make the measurements? *

- Bog
- Forest
- Glacier
- Mountain
- Lake Ice
- Plain (flat open area)
- Sea Ice
- Tundra
- Urban Area
- Other:

**Protocols of measured snow parameter**

Did you apply a written protocol (published or unpublished, written in any language) when measuring the snow parameters? *

- Yes, for all the measured parameters I applied a written protocol, which describes both the use of the instrumentation and its set up in various environmental conditions
- Yes, for some of the measured parameters I applied a written protocol, which describes both the use of the instrumentation and its set up in various environmental conditions. For some parameters I only followed the general instructions of the instrument and applied measurement strategy based on my own experience or on the oral teaching of senior scientists/operators
- No, I only followed the general instructions of the instrument and applied measurement strategy based on my own experience or on the oral teaching of senior scientists/operators.
- No, I don’t use any written protocol, I apply the instrument’s instructions using the instrument as I learned by myself.
- Other:

**Usage of measured snow parameter**

For what purpose the snow parameters are measured? *

- Research
- Operational

For which application the snow parameters are measured? *

- Agriculture and Forestry
- Avalanche
- Climatology
- Flooding
- Health and Sport
- Hydrology
- Meteorology
- Traffic
- Water Management
- Other:

## Appendix D. European Countries and Institutions Participating to the Survey

**Table A7:** List of European countries participating in the survey, with the total number of answers per country (second column), and the number of answers per institution (third column). The names of the institutions are provided in English. For clarity, the names of the institutions in the original languages (or their acronym) are provided in parenthesis for those cases in which the English translation is not commonly applied or the original acronym is widely known.

Country	Total N° of Answers	Number of Answers per Institution
**Albania**	1	1, Institute of GeoSciences, Energy, Water, and Environment (IGJEUM)
**Andorra**	1	1, Snow and Mountain Research Centre
**Austria**	4	2, Central Institution for Meteorology and Geodynamics (ZAMG) 1, University of Graz 1, Avalanche warning Centre Tirol (LWD)
**Belgium**	2	1, University of Leuven (KU Leuven) 1, Royal Meteorological Institute
**Bosnia and Herzegovina**	1	1, Federal hydrometeorological institute
**Bulgaria**	3	3, National Institute of Meteorology and Hydrology
**Croatia**	1	1, Croatian Meteorological and Hydrological Service (DHMZ)
**Cyprus**	1	1, Cyprus Department of Meteorology
**Czech Republic**	3	1, Czech University of Life Sciences, Department of Agroecology and Biometeorology 1, Czech Hydrometeorological Institute (CHMI) 1, Charles University, Department of Physical Geography and Geoecology
**Denmark**	2	1, Danish Meteorological Institute (DMI) 1, Geological Survey of Denmark and Greenland (GEUS)
**Estonia**	2	2, Estonian Environment Agency
**Finland**	12	8, Finnish Meteorological Institute (FMI) 2, Finnish Geospatial Research Institute 1, Finnish Environment Institute (SYKE) 1, University of Oulu
**France**	9	1, National Center for Scientific Research/Center for the Study of the Biosphere from Space (CNRS/CESBIO) 1, National Center for Scientific Research/Laboratory of Hydrology and Environment Transfers (CNRS/LTHE) 2, Météo-France 2, Laboratory of Glaciology and Environmental Geophysics (LGGE) 1, Grenoble Alps University 1, National Research Institute of Science and Technology for Environment and Agriculture (IRSTEA) 1, French Electricity (Electricité de France—EDF)
**Germany**	3	1, Alfred Wegener Institute 1, German Weather Service (DWD) 1, Ludwig-Maximilian University of Munich, Depart. Earth and Environmental Sciences
**Greece**	1	1, University of Thessaly
**Hungary**	3	3, Hungarian Meteorological Service (OMSZ)
**Iceland**	3	1, Icelandic Meteorological Office 1, Reykjavik University 1, National Power Company of Iceland (Landsvirkjun)
**Ireland**	2	1, University College Dublin 1, Irish Meteorological Service
**Italy**	12	3, Regional Environmental Protection Agency (ARPA) 1, National Research Council/Institute for the Dynamics of Environmental Processes (CNR/IDPA) 1, University of Pavia 1, Insubria University 1, Civil protection of Friuli Venezia Giulia Region 1, Civil Protection of Marche Region 1, Civil protection of Bolzan Province 2 Snow and Avalanche Warning Service of Valle d’Aosta 1, Italian Air Force/Technical Centre for Meteorology
**Latvia**	1	1, Latvian Environment, Geology and Meteorology Centre
**Lithuania**	2	1, Lithuanian Hydrometeorological Service 1, Vilnius University
**Luxembourg**	1	1, Agricultural Service (ASTA)
**Norway**	3	2, Norwegian Water Resource and Energy Directorate (NVE) 1, University of Oslo
**Poland**	6	1, Institute of Meteorology and Water Management—National Research Institute (IMGW-PIB) 1, University of Silesia 2, Institute of Geophysics-Polish Academy of Sciences 1, Nicolaus Copernicus University in Toruń, N.Copernicus Polar Station on Spitsbergen 1, University of Wrocław, Department of Climatology and Atmosphere Protection
**Portugal**	1	1, Portuguese Institute of the Sea and the Atmosphere (IPMA)
**Republic of Macedonia**	1	1, Hydrometeorological Service
**Romania**	1	1, National Meteorological Administration
**Russia**	3	2, Russian State Hydrometeorological University (RSHU) 1, Federal State Budget Institution—North-West Management of Hydrometeorology and Environmental Monitoring (FGBU—Severo-Zapadnoe UGMS)
**Serbia**	1	1, Republic Hydrometeorological Service of Serbia
**Slovakia**	3	1, Earth Science Institute 1,Technical University in Zvolen (TU Zvolen) 1, Institute of Hydrology—Slovak Academy of Sciences
**Slovenia**	7	1, Slovenian Environment Agency 1, Slovenian Forestry Institute 1, Geodetic Institute of Slovenia 1, Scientific Research Centre of the Slovenian Academy of Sciences and Arts, Anton Melik Geographical Institute 1, Jožef Stefan Institute 1, Anton Melik Geographical Institute (ZRC SAZU) 1, Mountain Rescue of Slovenia (Gorska reševalna zveza Slovenije)
**Spain**	8	1, Spanish National Research Council (CSIC) 1, Spanish State Meteorology Agency (AEMET) 1,University of Salamanca 1, Spanish Geological Survey (IGME) 1,University of the Basque Country 1,University of Granada 1, General Water Directorate 1, Environment and Water Agency of Andalusia
**Sweden**	3	2, Swedish Meteorological and Hydrological Institute (SMHI) 1, Water regulation enterprises (Vattenregleringsföretagen)
**Switzerland**	5	2 WSL Institute for Snow and Avalanche Research (SLF) 1University of Bern—Institute of Geography 1, Meteo Swiss 1, University of Zurich
**The Netherlands**	1	1, Institute for Marine and Atmospheric research Utrecht, Utrecht University
**Turkey**	4	1, Middle East Technical University (METU) 1, Anadolu University 1, Turkish State Meteorological Service 1, General Directorate of State Hydraulic Works (DSI)
**United Kingdom**	7	3, Met Office 1, Scottish Environment Protection Agency 1, Scottish Avalanche Information Service 1, University of Edimburg 1, Northumbria University
**Ukraine**	1	1, Ukrainian Hydrometeorological Institute
